# CRISPLD1: a novel conserved target in the transition to human heart failure

**DOI:** 10.1007/s00395-020-0784-4

**Published:** 2020-03-07

**Authors:** Sara Khadjeh, Vanessa Hindmarsh, Frederike Weber, Lukas Cyganek, Ramon O. Vidal, Setare Torkieh, Katrin Streckfuss-Bömeke, Dawid Lbik, Malte Tiburcy, Belal A. Mohamed, Stefan Bonn, Karl Toischer, Gerd Hasenfuss

**Affiliations:** 1 Laboratory of Experimental Cardiology, Clinic for Cardiology and Pneumology, Heart Research Center, University Medical Center Goettingen, Robert-Koch-Str. 40, 37075 Göttingen, Germany; 2 German Centre for Cardiovascular Research (DZHK), partner site Goettingen, Clinic for Cardiology and Pneumology, Heart Research Center, University Medical Center Göttingen, Robert-Koch-Str. 42a, 37075 Göttingen, Germany; 3 Stem Cell Unit, Clinic for Cardiology and Pneumology, University Medical Center Goettingen, Göttingen, Germany; 4 German Center for Neurodegenerative Diseases (DZNE) Goettingen Site, Göttingen, Germany; 5 Scientific Genomics Platform, BIMSB, Max Delbrück Center for Molecular Medicine, Berlin, Germany; 6 Institute of Pharmacology and Toxicology, University Medical Center Goettingen, Göttingen, Germany; 7 Center for Molecular Neurobiology Hamburg, Institute of Medical Systems Biology, University Medical Center Hamburg-Eppendorf, Hamburg, Germany; 8 German Center for Neurodegenerative Diseases (DZNE), Tübingen, Germany

**Keywords:** Heart failure, Compensated hypertrophy, Calcium cycling, iPSC-CM

## Abstract

Heart failure is a major health problem worldwide with a significant morbidity and mortality rate. Although studied extensively in animal models, data from patients at the compensated disease stage are lacking. We sampled myocardium biopsies from aortic stenosis patients with compensated hypertrophy and moderate heart failure and used transcriptomics to study the transition to failure. Sequencing and comparative analysis of analogous samples of mice with transverse aortic constriction identified 25 candidate genes with similar regulation in response to pressure overload, reflecting highly conserved molecular processes. The gene cysteine-rich secretory protein LCCL domain containing 1 (CRISPLD1) is upregulated in the transition to failure in human and mouse and its function is unknown. Homology to ion channel regulatory toxins suggests a role in Ca^2+^ cycling. CRISPR/Cas9-mediated loss-of-function leads to dysregulated Ca^2+^ handling in human-induced pluripotent stem cell-derived cardiomyocytes. The downregulation of prohypertrophic, proapoptotic and Ca^2+^-signaling pathways upon CRISPLD1-KO and its upregulation in the transition to failure implicates a contribution to adverse remodeling. These findings provide new pathophysiological data on Ca^2+^ regulation in the transition to failure and novel candidate genes with promising potential for therapeutic interventions.

**Electronic supplementary material:**

The online version of this article (10.1007/s00395-020-0784-4) contains supplementary material, which is available to authorized users.

## Introduction

Systolic heart failure (HF) is a leading cause of hospital admission and mortality in industrialized nations. Despite extensive efforts, progress in the development of new specific drugs is lacking [39]. Hence, there is significant interest in developing therapies that block or reverse the deleterious effects of prolonged cardiac stress.

The first response of the heart upon a cardiac insult (such as hypertension, valvular stenosis or insufficiency) is a compensated hypertrophy (CH) with preserved ejection fraction (EF) and cardiac function. This adaptive response is accompanied by molecular changes that impair contractility over time leading to “decompensated” HF with decreased cardiac function and left ventricular dilation [16, 26, 47, 49]. Life-threatening symptoms such as mechanical failure and arrhythmias in later stages lead to the poor outcome of this complex disease [33]. The terminal “end stage” of HF (tHF) has been analyzed using myocardium samples from heart transplantation surgeries [3, 44, 48, 53]. However, due to the unavailability of patient samples, there is a lack of data regarding the molecular transition from health to CH and moderate HF (mHF). Primary sources for the analysis of progression steps during disease development are animal models. However, despite a common phenotype, results gained from animal models cannot necessarily be transferred to the human situation [8, 11, 23]. RNA-sequencing (RNA-seq) screens in a variety of animal HF models and human tHF showed divergence in mRNA expression profiles [1, 2, 5, 28, 42, 45]. Hence, adequate models for understanding the transcriptional basis of HF disease progression are missing.

To identify novel functional target genes in the human heart during disease progression in response to pressure overload (PO), we carried out an RNA-seq screen in human myocardium of aortic stenosis (AS) patients in comparison with control myocardium from potential organ donors. Aortic valve replacement procedure enabled to collect myocardium samples of patients with AS and CH and normal EF and mHF with reduced EF. To compare the human data to the mouse model of PO-induced CH and mHF, we conducted RNA-seq at similar stages in the transverse aortic constriction (TAC) mouse model. The direct comparison of human and mouse gene expression profiles led to the identification of 25 genes regulated analogously in the human and the mouse heart in response to PO.

The candidate gene cysteine-rich secretory protein LCCL domain containing 1 (CRISPLD1) is secreted and was previously found in the secretome of choroid plexus epithelial cells [29] and exosomes of human parotid gland [19] and prostatic secretions [40]. There is no literature available on either human or mouse CRISPLD1 function. However, CRISPLD1 contains allergen V5/Tpx-1-related conserved sites, which have been shown to be evolutionarily related to helothermine, a toxin found in the venom of the Mexican beaded lizard (*Heloderma horridum horridum*) that blocks cardiac ryanodine receptor (RyR) channels and Ca^2+^-induced Ca^2+^ release (CICR) [32, 34, 35].

Ca^2+^ plays a central role in the heart and in HF development [20, 22, 31]. Remodeling of Ca^2^ handling accounts to contractile dysfunction and arrhythmogenesis, responsible for the detrimental outcome of HF [27]. CRISPLD1 upregulation during the transition to HF and its sequence homology to toxins such as helothermine implicated a functional role in regulation of Ca^2+^ cycling and thus cardiac contraction during disease development. Therefore, we investigated the role of CRISPLD1 by CRISPR/Cas9-mediated loss-of-function experiments in human-induced pluripotent stem cell-derived cardiomyocytes (hiPSC-CM). Confocal Ca^2+^ imaging of hiPSC-CM show an increase in Ca^2+^ transient (CaT) amplitude, CaT rise time and a faster CaT decay after CRISPLD1 knock out (CRISPLD1-KO). Accordingly, this indicates that CRISPLD1, a previously uncharacterized secretory protein, plays an inhibitory role in Ca^2+^ handling in human CM and might have a detrimental effect on regulation of Ca^2+^cycling during disease development. Analysis of the CRISPLD1-KO transcriptome and proteome and CRISPLD1 upregulation during HF progression suggests a contribution to the failing phenotype of the heart. Thus, deep RNA-seq in patients at the transition to HF identified the novel candidate gene CRISPLD1 as a regulator of Ca^2+^ cycling.

## Results

### Transcriptomics in human transition from CH to mHF and TAC-mice

**Fig. 1:**
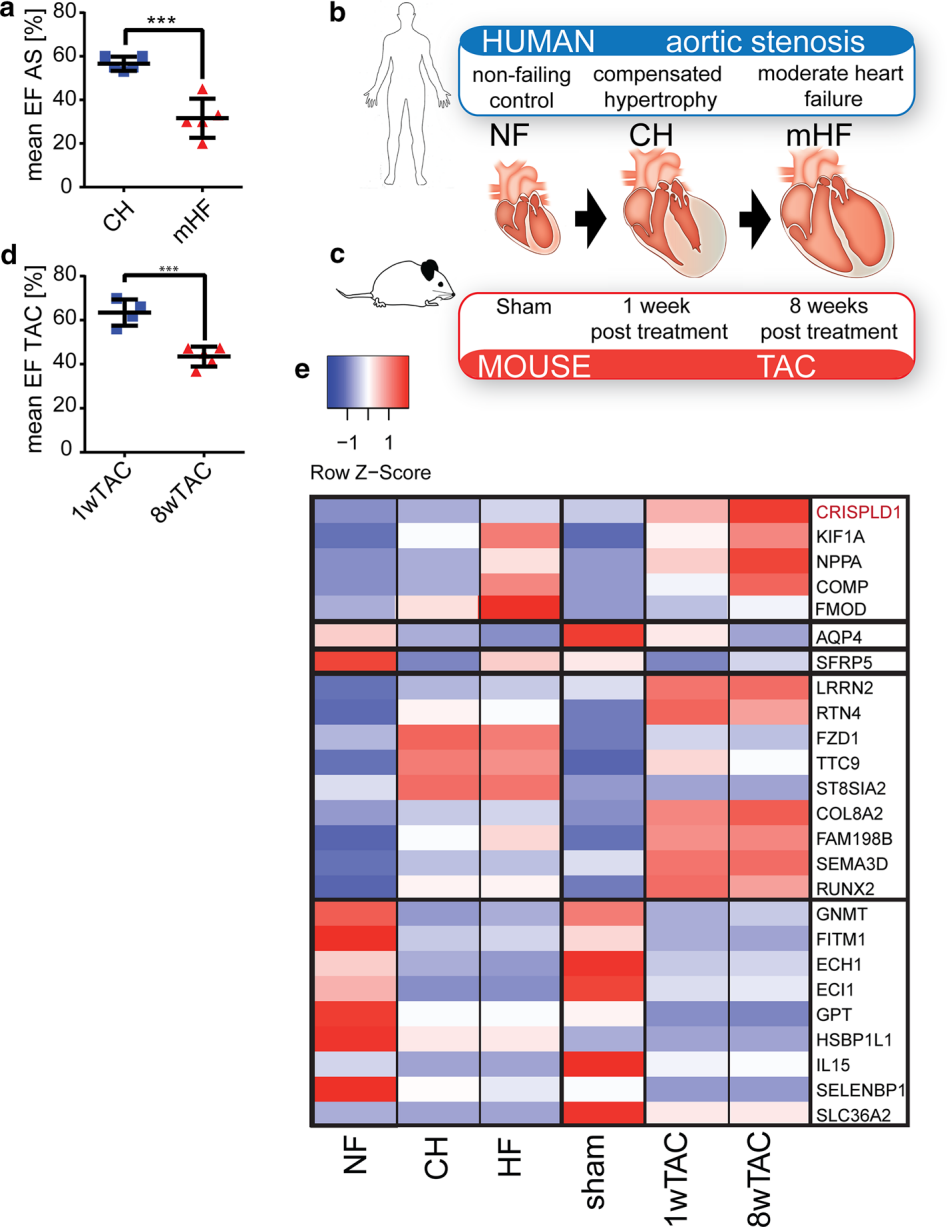
RNA-seq of PO-induced CH and mHF in human and mouse myocardium. **a** EF of AS patients with CH and mHF. Error bars represent mean ± SEM. **b** Workflow of RNA-seq screen of human NF samples (*n* = 4), human AS patients with CH (*n* = 5) and mHF (*n* = 5) and **c** of mice 1 week (CH) (*n* = 4) and 8 weeks (mHF) after TAC (*n* = 5). Error bars represent mean ± SD. **d** EF of mice 1wTAC and 8wTAC. Error bars represent mean ± SEM. **e** Expression profiles of conserved DEGs in human and mouse transcriptomes during disease progression illustrated as heatmap. The light blue to dark red shading scale reflects the logFC of median normalized counts. Genes expressed in the same way in human and mouse datasets according to disease progression are framed. CRISPLD1 is highlighted in red. *EF *ejection fraction, *AS *aortic stenosis, *CH *compensated hypertrophy, *mHF *moderate heart failure, *NF *non-failing control, *1wTAC *1 week post-transverse aortic constriction, *8wTAC *8 weeks post-transverse aortic constriction, *DEGs *differentially expressed genes, *logFC *log fold change. *n* = 4–5/group

Biopsy samples from human myocardium were obtained from aortic valve replacement surgery (see suppl. Table 1 for clinical characteristics). Figure 1a, b details the samples that were used for sequencing. The following groups were compared: a non-failing (NF) control group with four left ventricular myocardial samples from healthy donor hearts and two pathology groups of AS patients, each consisting out of five replicates and being matched for age and sex. A terminal heart failure group (tHF) with six left ventricular myocardial samples from transplanted hearts of patients suffering dilated cardiomyopathy (DCM) was used as out-group (see suppl. data file S1 for differential gene expression data). The major difference between the two AS pathology groups is the ejection fraction (EF), which is preserved with values over 55% (58.4% ± 2.9) in the CH group and reduced with values of about 33% (33.5% ± 4.1) in the mHF group (graph Fig. 1a and suppl. Table 1).

To compare the human data to the mouse model of PO-induced CH and HF, we conducted RNA-seq at similar stages in the TAC mouse model using exactly the same methodology as for the human samples (Fig. 1c, see suppl. Fig. S1 for mouse echocardiography/morphometry). CH was compared to animals 1 week post-treatment (PT) (1wTAC) showing a mean preserved EF of 63% (± 2.7). mHF was compared to mice 8 weeks PT (8wTAC). 8wTAC animals show a significant reduction in EF (47% ± 2.2) comparable to the human situation. With this approach, we received for the first time a highly comparative dataset which allowed us to set up expression profiles of transcriptomic changes at different stages of PO in human AS patients (suppl. data S1) and mice after TAC (suppl. methods, suppl. data S2, suppl. Fig. S2). Principle component analysis (PCA) of the NF, CH mHF and tHF groups showed a low variation within the groups but allow a clear distinction due to high variation between NF, CH mHF and tHF (suppl. Fig. S3). PCA of biological mouse replicates also showed a clear distinction between control (sham) and treatment (TAC) groups (suppl. Fig. S4). The expression of known marker genes for CH and HF was validated by qPCR (suppl. Fig. S5). NPPB increases dramatically in expression in mHF compared to the control and MEF2A and MYH7 increase significantly in both conditions compared to the control.

We identified 25 genes that are regulated in a conserved manner in human and mouse HF progression (Fig. 1e) using highly stringent filtering (suppl. methods). Literature research was conducted to verify CH and HF pathology-related functions of the identified candidates using the available public databases (suppl. Table 2). Among the genes that are progressively upregulated in the transition to HF is CRISPLD1, a gene of unknown biological function (Fig. 1e and suppl. Table 2). Transcriptomics in human and mice led to the identification of candidate genes with a potential functional role in the transition process and more specifically the previously uncharacterized CRISPLD1 gene.

### CRISPLD1: a novel uncharacterized candidate in the transition to HF

Based on the expression profiles during HF progression in human and mouse transcriptomes and after extensive literature search, we decided to functionally test the role of the gene cysteine-rich secretory protein LCCL domain containing 1 (CRISPLD1).

**Fig. 2:**
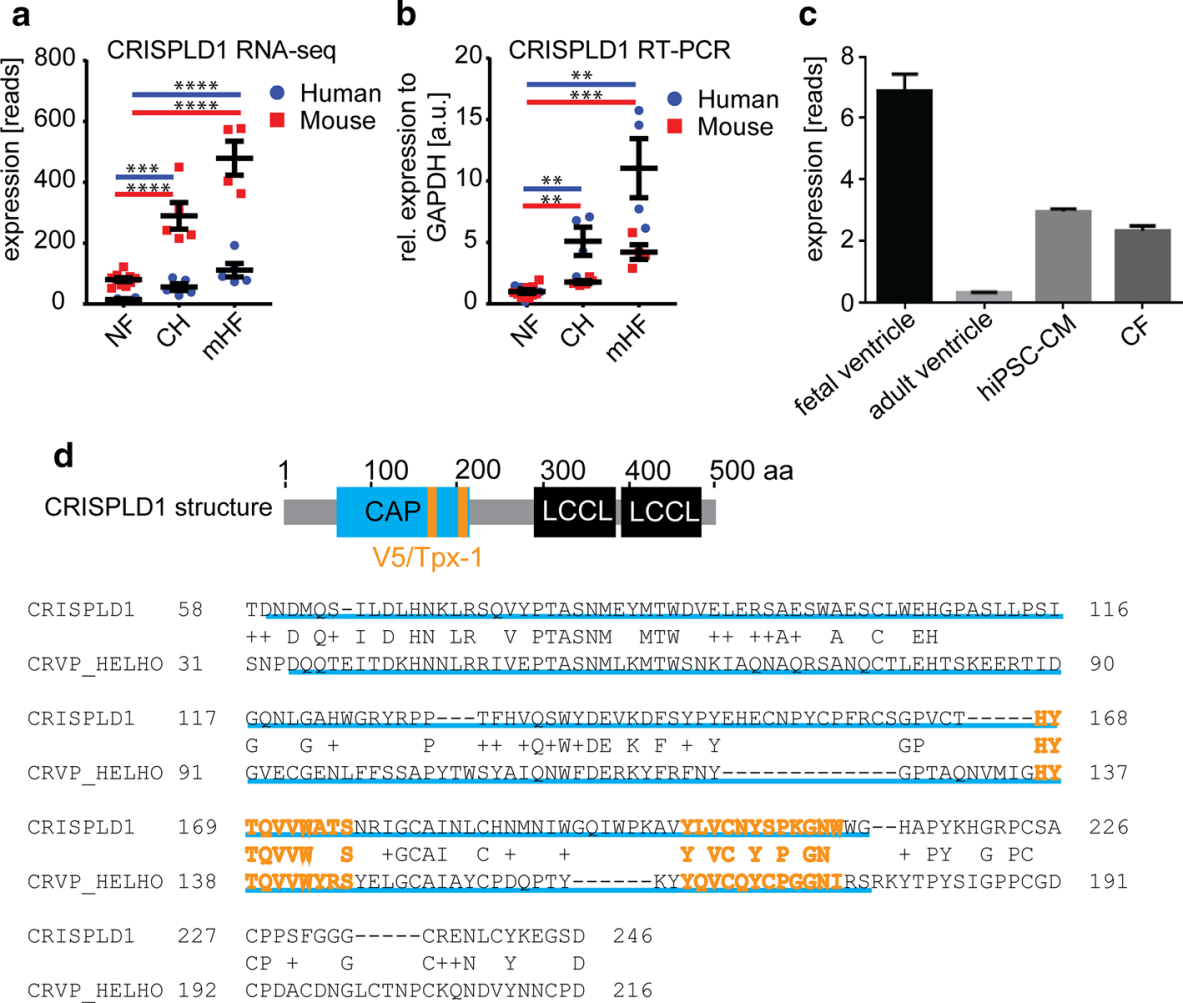
Expression profile, qPCR validation and protein structure of CRISPLD1**. a** Expression profile of CRISPLD1 in NF, CH and mHF determined by RNA-seq. Given are the reads (rpm) of AS patients and mice (sham = NF, 1wTAC = CH and 8wTAC = mHF); asterisks represent FDR-corrected *p *values. **b** qPCR validation of CRISPLD1 expression profiles in human and mouse NF (= human non-failing myocardium or mice sham non-failing myocardium), CH and mHF myocardium. **c** Expression of CRISPLD1 determined by RNA-seq (given as rpkm) in human fetal ventricle, adult ventricle, hiPSC-CM and cardiac fibroblasts (CF). **d** Protein structure of CRISPLD1 showing the CAP domain (155 aa, highlighted in blue) and two V5/Tpx-1 conserved sites (10aa and 12 aa, respectively, highlighted in orange. The two LCCL domains at the C-terminus are highlighted in black. The alignment shows conservation of CRISPLD1 and the cysteine-rich venom protein helothermine (CRVP_HELHO) protein sequence within the CAP domain (underlined in blue) and the two V5/Tpx-1 sites (orange letters, CRISPLD1 position 167–177 CRISP family signature 1; CRISPLD1 position 201–212 CRISP family signature 2). Alignment was generated using blastp. Error bars represent mean ± SEM. *rel. *relative, *a.u. *arbitrary units, *EF *ejection fraction, *NF *non-failing control, *AS *aortic stenosis, *CH *compensated hypertrophy, *mHF *moderate heart failure, *NF *non-failing control, *hiPSC-CM *human-induced pluripotent stem cell-derived cardiomyocytes. *n* = 4–5/group

CRISPLD1 is expressed at low levels in human and mouse NF myocardium, increases significantly in CH (log2foldchange = 1.6) and 1wTAC (log2foldchange = 1.5) and increases once more in mHF (log2foldchange = 2.6) and 8wTAC (log2foldchange = 2.3) (Fig. 2a). The sequencing results were validated by qPCR (Fig. 2b) and we also find CRISPLD1 to be expressed in fetal ventricle in hiPSC-CM and human cardiac fibroblasts (Fig. 2c).

The biological function of CRISPLD1 in the heart is to our knowledge not described in the existing literature, neither in human nor in mouse. Intriguingly, this secretory protein belongs to the highly conserved CRISP/Antigen 5/PR-1 (CAP) superfamily and contains (beside the CAP domain and two LCCL domains) V5/Tpx-1-conserved sites, which have been shown to be evolutionarily related to Ca^2+^channel regulating toxins such as the lizard toxin helothermine (Fig. 2d). Alignment of the CRISPLD1 protein sequence to the cysteine-rich venom protein helothermine (Fig. 2d) shows sequence conservation within the CAP domain and a high degree of conservation between the V5/Tpx-1 CRISP family signature 1 (position 167–177 of CRISPLD1, 80% identity) and signature 2 (position 201–212 of CRISPLD1, 60% identity).

**Fig. 3:**
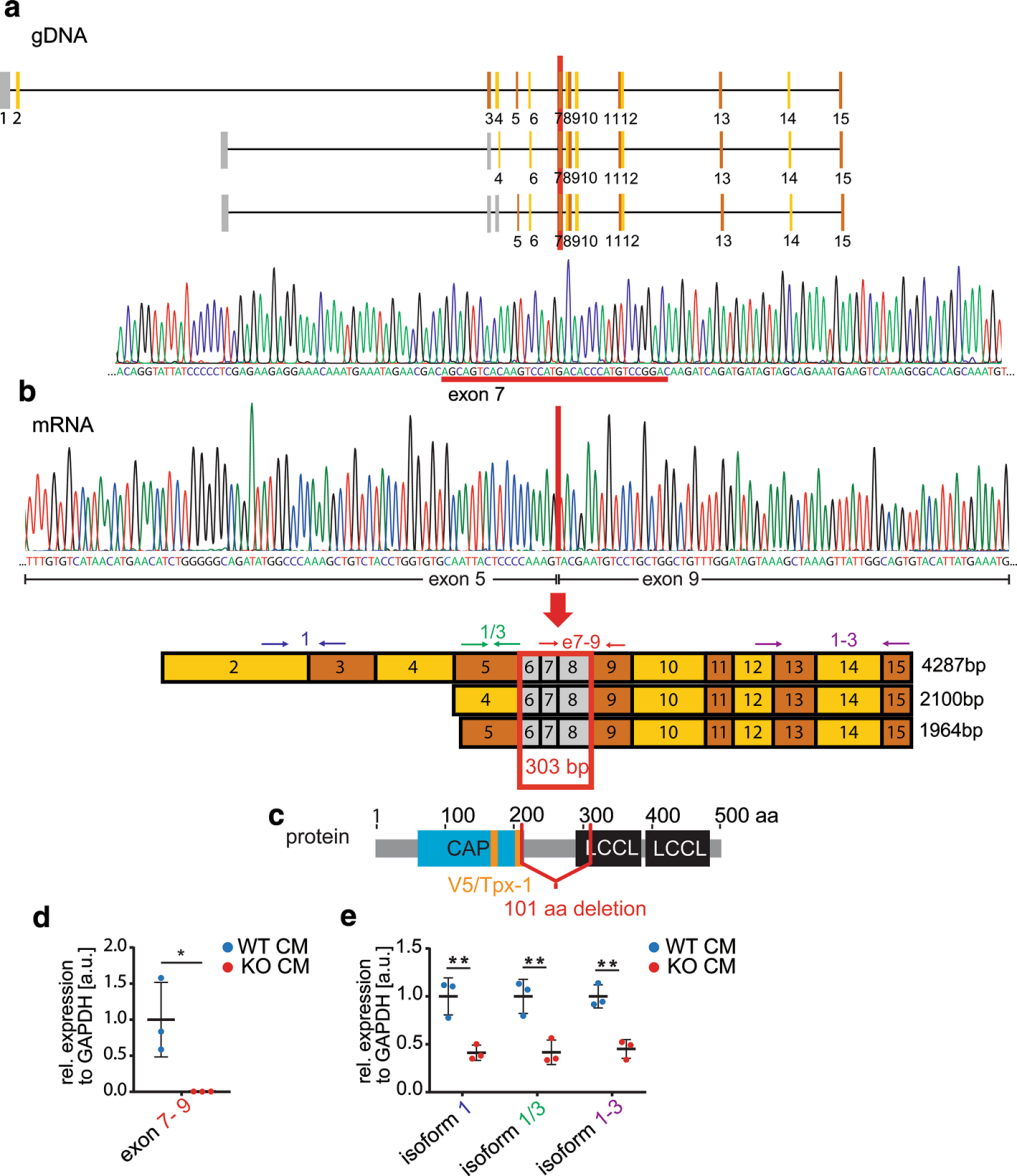
Structure of CRIPLD1-KO. **a** A 34 bp genomic DNA (gDNA) deletion located in exon 7 was introduced via CRISPR/CAS9. **b** Exon skipping leads to a loss of 303 base pairs (bp; exon 6–8) at mRNA level, causing a deletion **c** of 101 aa (amino acids, highlighted in red), affecting the functional domains of the protein. **d** qPCR of the deleted region on mRNA level in CRISPLD1-KO-CM and WT controls. **e** qPCR showing a significant reduction of the mRNA of all isoforms. Primer locations are indicated as arrows in **(b)**. Error bars represent mean ± SEM. *WT-CM *wild-type control hiPSC-CM, *KO-CM *CRISPLD1-KO-hiPSC-cardiomyocytes, *rel. *relative, *a.u. *arbitrary units; each data point reflects a mean of 3 technical qPCR replicates of *n* = 3 independent differentiations/group

Therefore, we decided to study the function of CRISPLD1 in a human CM model system with respect to the hypothetical Ca^2+^ regulating function. To get insights into CRISPLD1 function in human iPSC-CM, we introduced a genomic deletion in hiPSC via CRISPR/Cas9. Genome editing generated a 34 bp deletion located in exon 7, affecting all the three CRISPLD1 isoforms (Fig. 3a). Due to exon skipping as a consequence of the deletion, 303 bp of the mRNA are lost during transcription, resulting in an in-frame deletion of 101 amino acids (aa), affecting the major functional domains of the protein including the V5/Tpx-1 sites (Fig. 3b, c and suppl. information S8 for sequence information).

Next, we validated the deletion on mRNA level in hiPSC-CM via qPCR. No expression was detectable using a primer pair that was designed to amplify the deleted region (Fig. 3d). qPCR and sequencing of amplicons with primer pairs spanning regions outside the deletion showed, that there is still CRISPLD1 mRNA detectable. However, this mRNA lacks sequence information within the CAP, V5/Tpx-1 and LCCL domains and is significantly down regulated in all the three isoforms compared to the control (Fig. 3e and suppl. information S8). Therefore, we assume a functional KO of the CRISPLD1 protein.

CRISPR/Cas9-mediated functional KO of CRISPLD1 in hiPSC allowed us to test the role of CRISPLD1 in calcium cycling (as indicated by its sequence similarity to previously described calcium regulators) in human cardiomyocytes.

### CRISPLD1-KO affects calcium cycling in hiPSC-CM

Next, we conducted cellular and functional analysis of CaT and by confocal calcium imaging. The CRISPLD1-KO and the corresponding isogenic WT hiPSC line were differentiated into mainly ventricular-like functional CM. WT- and CRISPLD1-KO-hiPSC-CM were cultured up to 60 days for adequate maturation.

**Fig. 4:**
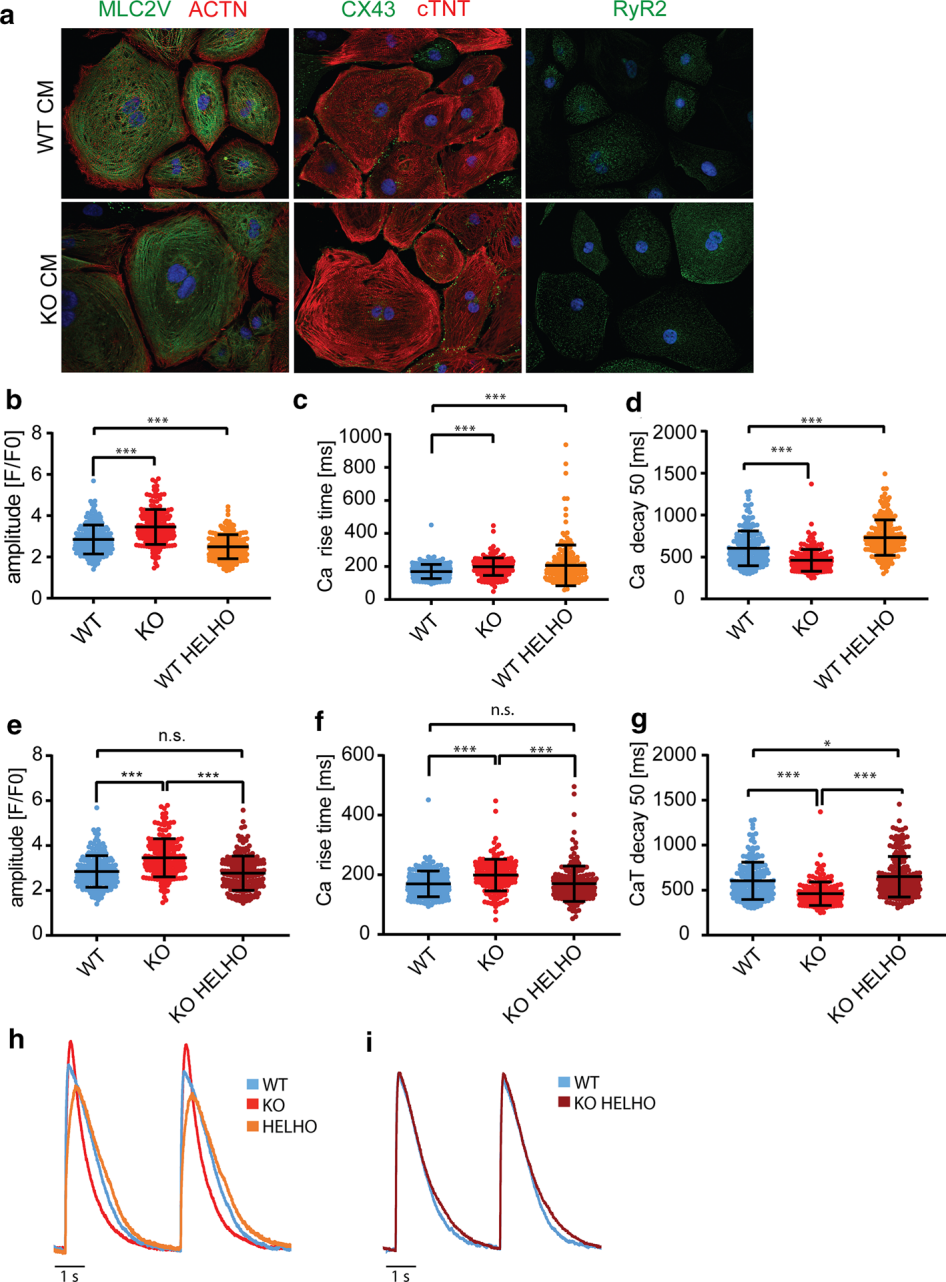
Characterization and functional analysis of CRISPLD1-KO-hiPSC-CM. **a** Representative images of immunofluorescence stainings of CM markers with anti-Myosin Light Chain 2 (MLC2V; green), anti-sarcomeric α-Actinin (ACTN; red), anti-cardiac Troponin T (cTNT; red), anti-Connexin 43 (CX43; green) and Anti-cardiac muscle ryanodine receptor (RyR2; green). Nuclei are shown in blue and stained with Hoechst33342. **b–i** Functional analysis of CRISPLD1-KO-hiPSC-CM at day 60–80 by confocal calcium imaging. **b** Ca^2+^ transient (CaT) amplitude **c** CaT rise time and **d** CaT decay at 50% of WT-hiPSC-CM, CRISPLD1-KO-hiPSC-CM and WT-hiPSC-CM treated with helothermine. **e** Ca^2+^ transient (CaT) amplitude **f** CaT rise time and **g** CaT decay at 50% of WT-hiPSC-CM, CRISPLD1-KO-hiPSC-CM and CRISPLD1-KO-hiPSC-CM treated with helothermine. **h** Overlay of representative single cell recordings of CaT of WT CM (blue), CRISPLD1-KO CM (red) and WT CM treated with helothermine (orange). **i** Overlay of representative single cell recordings of CaT of WT (blue) and CRISPLD1-KO CM treated with helothermine (purple). Error bars represent mean ± SD. *hiPSC-CM *human-induced pluripotent stem cell-derived cardiomyocytes, *WT *wild-type control, *KO *knock out, *WT HELHO *wild-type control treated with helothermine, *KO HELHO *knock out treated with helothermine, *HELHO *helothermine, *s *second. *n* = 181 cells (WT); *n* = 183 cells (KO)

CRISPLD1-KO showed no obvious effects on differentiation efficiency and contractile function compared to WT-hiPSC-CM. Immunofluorescence analysis of KO-hiPSC-CM compared to WT-hiPSC-CM (Fig. 4a) showed normal cardiac marker expression. Cardiac contraction filaments were stained with anti-α-actinin (ACTN) and cardiac troponin (cTNT). The ventricular isoform of the myosin light chain 2 (MLC2V) antibody was used to determine the ventricular identity of CM. The expression of ACTN and cTNT displayed regular organization of cardiac sarcomeres. There were no noticeable alterations in the expression patterns of MLC2V, cTNT and the cardiac-specific gap junction protein CX43. Furthermore, RyR2 was detectable in a CM-specific expression pattern in both WT-hiPSC-CM and KO-hiPSC-CM.

As we hypothesize a functional role of CRISPLD1 in calcium cycling, we carried out CaT analysis by confocal calcium imaging of CRISPLD1-KO-CM compared to WT-control-CM (Fig. 4b—i). Compared to WT controls, CRISPLD1-KO-CM show a significant increase in CaT amplitude (WT mean = 2.8, KO mean = 3.5; Fig. 4b, e), a significant increase in CaT rise time (WT mean = 169.5 ms, KO mean = 199.0 ms; Fig. 4c, f) and a significant decrease in CaT decay (WT mean = 603.5 ms, KO mean = 460.8 ms; Fig. 4d, g), pointing towards higher systolic CaT in KO-CM.

To further validate the calcium-imaging data and to test the effect of CRISPLD1-KO upon toxin treatment with a known Ca^2+^ regulator, we treated WT-control-CM and CRISPLD1-KO-CM with helothermine. WT-control-CM exhibit a significantly decreased CaT amplitude upon helothermine treatment compared to WT controls without treatment (WT mean = 2.8, KO mean = 3.5, WT HELHO mean = 2.5; Fig. 4b, h). The CaT rise time is significantly increased in WT-CM treated with helothermine compared to WT-controls (WT mean = 169.5 ms, KO mean = 199.0 ms, WT HELHO = 207.1 ms; Fig. 4c, h). The CaT decay is significantly increased upon helothermine treatment compared to WT controls without treatment (WT mean = 603.5 ms, KO mean = 460.8 ms, WT HELHO mean = 731. 9 ms; Fig. 4d, h). CRISPLD1-KO-CM do not show a significant alteration in CaT amplitude after helothermine treatment compared to WT-control-CM. Instead, the CaT amplitude is of normal WT range (WT mean = 2.9, KO mean = 3.5, KO HELHO mean = 2.8; Fig. 4e, i). This is also true for the CaT rise time: the significant increase observed in WT helothermine-treated CM is not detectable in CRISPLD1-KO-CM treated with helothermine and is rather comparable to WT controls (WT mean = 169.5 ms, KO mean = 199.0 ms, KO HELHO mean = 170.0 ms; Fig. 4f, i). The increased CaT decay in WT helothermine-treated CM is still detectable in the CRISPLD1-KO-CM line treated with helothermine, but to a much lower degree (WT mean = 603.5 ms, KO mean = 460.8 ms, KO HELHO mean = 650.2 ms; Fig. 4g, i).

We were able to detect a functional, measurable effect on CaT in CRISPLD1-KO-CMs (increased Ca^2+^ amplitude and Ca^2+^rise time and decreased Ca^2+^decay). CRISPLD1-KO is able to rescue the CaT phenotype produced by treatment with the Ca^2+^ regulating toxin helothermine (normalization of Ca^2+^ amplitude and Ca^2+^rise time). This loss-of-function and rescue experiments support the converse argument, that CRISPLD1 plays an inhibitory role in CM Ca^2+^ cycling.

### Transcriptomics reveal downregulation of WNT-, apoptosis-, TGF-beta and calcium signaling in response to CRISPLD1-KO

**Fig. 5:**
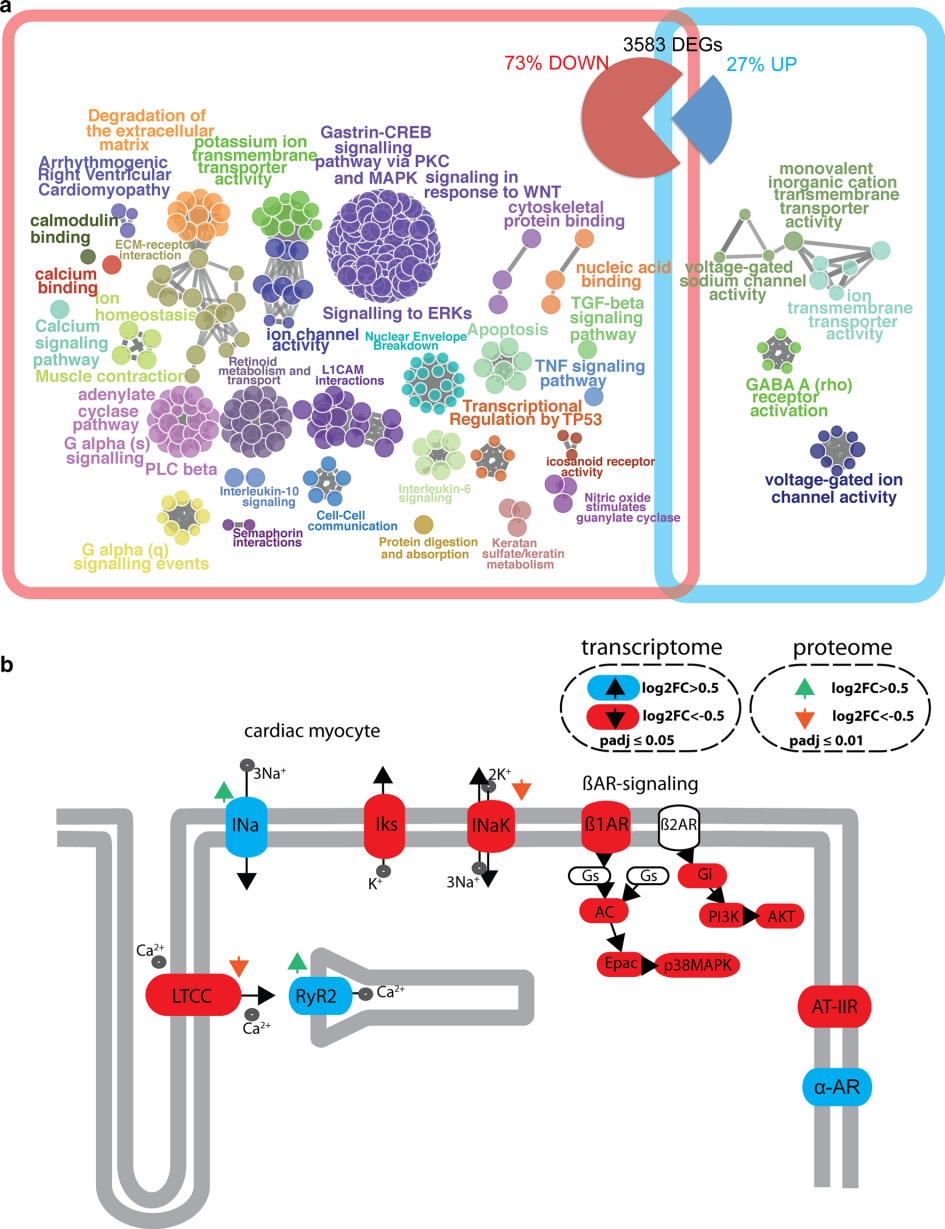
Transcriptomics and pathway analysis of CRISPLD1-KO-hiPSC-CM. **a** RNA-seq of CRISPLD1-KO-CM resulted in the identification of 3583 differentially expressed genes (DEGs) compared to WT-hiPSC-CM at day 60 (log_2_ Fold Change > 0.5/< − 0.5, adjusted *p *value < 0.05). 73% of the DEGs are downregulated and 27% are upregulated. Analysis of functionally organized gene ontology (GO)/pathway term network of down—(red square) and upregulated (blue square) significantly associated DEGs. The network represents GO/functional terms as nodes, which are linked based on a predefined kappa score level. The size of the nodes reflects the enrichment significance of the terms. Functional groups are colored and overlaid with the network. Functional groups are represented by their most significant (leading) term shown next to the corresponding group. **b** Simplified adrenergic signaling pathway in cardiomyocytes (pathway map adapted from KEGG entry hsa04261). Significantly regulated components are highlighted (transcriptome: upregulated = blue, downregulated = red; proteome: upregulated = green arrow, downregulated = orange arrow). LTCC = L-type calcium channel Iks = potassium voltage-gated channel INaK = Na + /K + -ATPase; *AC*  adenylate cyclase, *Epac * Rap guanine nucleotide exchange factor, *p38MAPK * mitogen-activated protein kinase 11, *Gi * G protein subunit alpha i2, *PI3K* phosphoinositide-3-kinase, *AKT * AKT serine/threonine kinase, *Ina * cardiac sodium channel, *RyR2 * cardiac ryanodine receptor, *ß1AR * adrenoreceptor beta 1, *AT-IIR * angiotensin II receptor type 1, *α-AR * adrenoceptor alpha

To get deeper insights into the biological function of CRISPLD1 in hiPSC-CM, samples from four independent differentiation experiments of WT control and CRISPLD1-KO-hiPSC-CM at day 60 (using three replicates for each differentiation) were sequenced for transcriptomics. PCA showed a clear distinction between control and KO replicates (suppl. Fig. 6) and differential expression analysis led to 3583 significantly regulated genes (suppl. data S3 and Fig. 5). 73% of the DEGs are downregulated in response to CRISPLD1-KO and 27% are upregulated (Fig. 5). Functional pathway and network analysis identified a highly interconnected functional network of GO/pathway terms, representing 85% of downregulated DEGs (Fig. 5 and suppl. data S4). Among the most significant downregulated pathways are apoptosis signaling, TGF-beta signaling pathway, TNF signaling pathway and transcriptional signaling by TP53 and signaling in response to WNT. Interestingly, genes associated to potassium ion transmembrane transporter activity, arrhythmogenic right ventricular cardiomyopathy, calcium signaling pathway, ion homeostasis and muscle contraction are functionally grouping, as well as genes associated to adenylate cyclase pathway, G alpha (s) signaling, PLC beta and G alpha (q) signaling. 76% of the upregulated genes are associated to pathway terms such as voltage-gated sodium channel activity, ion transmembrane transporter activity and inorganic cation transmembrane transporter activity (suppl. data S5).

As these pathways relate to the functional changes, which we observed by CaT analysis in CRISPLD1-KO-CM, we visualized the changes in gene expression in CM adrenergic signaling using KEGG search and color pathway (summarized in Fig. 5b, for detailed analysis, see suppl. Fig. S7a and suppl data S6). Representative gene expression changes observed by RNA-seq were validated by qPCR (suppl. Fig. S7b). The detailed analysis of the adrenergic signaling pathway in human CM shows a significant downregulation of ADRB1 mRNA (encoding adrenoceptor beta 1). Transcripts of ßAR-signaling downstream targets (genes encoding AC, Epac, p38MAPK, Gi, PI3K, AKT, CREB) and angiotensin II receptor type 1 (AGTR1) are downregulated as well. Among upregulated genes are ADRA1B, the RYR2 gene as well as genes encoding the cardiac sodium channel (SCN5A, SCN4B), while genes encoding the potassium channel (KCNE1), the catalytic subunit alpha-2 of the sodium–potassium ATPase (ATP1A2) and the l-type calcium channel (LTCC, CACNA2D2) are downregulated. To validate these findings, we carried out proteomics of WT control and CRISPLD1-KO-hiPSC-CM at day 60. Analysis of the liquid chromatography–tandem mass chromatography (LC–MS/MS) data revealed an upregulation of the cardiac ryanodine receptor (RYR2) and the cardiac sodium channel (SCN5A) on protein level as well as downregulation of the LTCC CACNAD3 protein and the ATPase Na + /K + transporting subunit alpha 3 (ATP1A3) (Fig. 5b and suppl data S7).

Thus, transcriptomics of human WT-CM and CRISPLD1-KO-CM led to the identification of regulated pathways specifically enriched upon CRISPLD1 loss-of-function. These pathways relate to the functional changes observed in the CaT analysis and deeper analysis of the adrenergic signaling pathway and proteome analysis support the functional role of CRISPLD1 in calcium cycling in human CM.

## Discussion

With a set of human myocardium biopsy samples of AS patients at two stages of HF progression, namely CH and mHF and healthy donor LV myocardium, we were able to conduct expression profiling in the human heart in response to PO. Analogous myocardium samples were obtained from TAC mice. We identified 25 genes with well-conserved expression profiles during HF progression in human and mouse. Among the candidates was a previously uncharacterized gene, CRISPLD1. We identified a novel functional role of CRISPLD1 in the regulation of Ca^2+^ handling in human CM and the transition to failure.

Using two time points, 1 week and 8 weeks post-TAC, we aimed to compare the human data to the TAC mouse model of PO-induced CH and HF. RNA-seq and the direct comparison of human and mouse gene expression profiles led to the identification of a set of 25 genes with analogous regulation in the human and the mouse heart in response to PO.

The direct stringent comparison of human and mouse transcriptomes shows a relative small overlap of molecular changes. This is, however, not surprising. Since different model systems and etiologies show divergent changes in the transcription profiles in response to HF [1, 2, 21, 28, 42]. The TAC surgery produces an acute PO as a result of aortic banding. On the contrary, human AS patients acquire valvular stenosis over a time span of years. The dataset presented here provides 25 conserved DEGs that are ideal candidates for further functional analysis. However, a limitation of this study is the number of myocardial AS samples available (five CH and five moderate HF), and corresponding four controls, which to some extent, limits the more exhaustive identification of a larger panel of potential differently expressed genes. Yet, the availability of human myocardial samples in general but specifically of non-failing control myocardium, patients with compensated hypertrophy and sustained cardiac function and moderate HF are scarce. Therefore, we focused our analysis on candidate genes with conserved regulation in the mouse model during the transition to heart failure. These genes reflect highly conserved molecular processes among species independent of the underlying etiology, which give rise to the PO-induced phenotype. Furthermore, the dataset enables comparative functional experiments in human and mouse model systems.

The candidate gene CRISPLD1 is encoding a protein that is functionally not described in the heart. It was previously found in the secretome of choroid plexus epithelial cells [29] and exosomes of human parotid gland [19] and prostatic secretions [40]. CRISPLD1 was described to be expressed in lung fibroblasts and was discussed as a novel biomarker for experimental pulmonary fibrosis [4]. It plays a role in face morphogenesis and the folic acid pathway [10, 51] and in chondrocyte response to IL-1α related to cellular stress [52]. CRISPLD1 was identified among regulators of murine hematopoietic stem cell repopulation [24]. It was also found to be regulated in a whole exome sequencing screen for cancer gene alterations by mTOR-activated tuberous sclerosis complex-associated renal cell carcinoma [38]. Only recently, it has been shown that CRISPLD1 polymorphisms alter antiplatelet potency of clopidogrel in coronary artery disease patients in Chinese Han [50] and that CRISPLD1 is differentially methylated during male infertility [46]. We focused our analysis on this gene, because it shows a very interesting protein structure. CRISPLD1 belongs to the highly conserved family of CRISP/Antigen 5/PR-1 (CAP) proteins. CAP superfamily proteins are most often secreted and the overall structural conservation was proposed to result in fundamentally similar functions for the CAP domain in all members [reviewed in [18]]. CAP proteins were shown to play a role in a number of diseases including HF. Of note, the CAP member peptidase inhibitor 16 (PI16) has been identified as a secreted protein involved in the negative regulation of CM size [17]. Loss of function of PI16 in CM led to increased cell size reorganized sarcomeres. Furthermore, PI16 was suggested to may have a role in matrix remodeling in HF. Uniquely among the CAP superfamily, the CRISPs have ion channel regulatory activity. This was first characterized in helothermine, a CRISP from the venom of the poisonous lizard Heloderma horridum horridum (Mexican beaded lizard) [32, 34, 35]. As CRISPLD1 shows high-sequence homology and has two V5/Tpx-1 sites related to CRISP1 and CRISP2 signatures (PROSITE patterns PS01009 and PS01010; see also InterPro database entry B7Z8V9 for CRISPLD1 domain architecture), we decided to study the function of CRISPLD1 in hiPSC-CM. We successfully generated a KO line in hiPSC via CRISPR/ CAS9, leading to the loss of parts of the CAP domain and V5/TPX-1-related site and to a significant downregulation of the remaining mRNA. Next, we examined the effect of CRISPLD1 loss-of-function on CM Ca^2+^ cycling. Quantifying CaT after CRISPLD1-KO compared to WT-CM showed a CM-specific function of CRISPLD1 in the regulation of Ca^2+^ cycling. CRISPLD1-KO-CM show a significant increase in CaT amplitude and rise time and a significant decrease in Ca^2+^ decay, pointing towards an increase in systolic CaT as a consequence of CRISPLD1 loss-of-function. In order to further strengthen our hypothesis and the data gained from CaT experiments, we carried out additional functional experiments to test, whether CRISPLD1-KO has the ability to rescue the effect of a known calcium regulator. We selected the lizard toxin helothermine as the ability of the secreted venom protein to block calcium cycling in many cell types is well described [32, 34, 35]. Helothermine treatment resulted in disturbed calcium transients in WT iPSC-CM with significantly decreased CaT amplitude and significantly increased CaT rise time and CaT decay. Indeed, CRISPLD1-KO is able to rescue the CaT phenotype produced by treatment with the Ca^2+^ regulating toxin helothermine (normalization of Ca^2+^ amplitude and Ca^2+^ rise time).

Eventually, we were able to detect a functional, measurable effect on CaT in CRISPLD1-KO-CMs (increased Ca^2+^ amplitude, Ca^2+^ rise time and Ca^2+^ decay). As the downregulation of CRISPLD1 mRNA leads to an increase in CaT, loss-of-function and rescue experiments support the converse argument, that CRISPLD1 plays a functional inhibitory role in human CM Ca^2+^ cycling. The progressive increase in CRISPLD1 expression during disease progression in AS patients points towards a functional role in the transition to HF, presumably as a negative regulator of Ca^2+^ cycling. However, the detailed underlying molecular mechanisms remain to be investigated in the future.

RNA-seq of CRISPLD1-KO-hiPSC-CM compared to WT-hiPSC-CM revealed a fundamental role of CRISPLD1 in human CM homeostasis. The downregulation of prohypertrophic and proapoptotic (G-protein signaling, CREB signaling, WNT signaling, TGF-beta signaling, TNF signaling, TP53 signaling) pathways (reviewed in [30]) points towards a beneficial downstream effect of CRISPLD1 loss-of-function on the human CM phenotype. The detailed analysis of the adrenergic signaling pathway in human CM supports this suggestion. Of note, the gene ADRB1, encoding the adrenoceptor beta 1, is significantly downregulated. ADRB1 is the predominant cardiac subtype expressed and known to confer increases in inotropy and chronotropy upon stimulation [9]. HF is associated to maladaptive chronic stimulation of adrenergic signaling, leading to detrimental effects on cardiomyocyte growth and function [7, 37]. One of the most common therapeutic approach in HF treatment using “beta-blockers” is targeting ADRB1 [43]. Furthermore, downstream targets of ßAR-signaling are downregulated (genes encoding AC, Epac, p38MAPK, Gi, PI3K, AKT, CREB). Another frequently used therapeutic strategy is the use of angiotensin-converting enzyme (ACE) inhibitors. Angiotensin II receptor type 1 (AGTR1) is significantly downregulated in the transcriptome of CRISPLD1-KO-CM. Previously, it has been shown that carriers with the mutation AGTR1 1166C experience a greater long-term compensatory renin–angiotensin–aldosterone system activation following treatment with candesartan  [13, 14]. Interestingly, the alpha1-adrenergic receptor (ADRA1B) previously shown to prevent a maladaptive cardiac response to PO [36] is upregulated after CRISPLD1 loss-of-function.

Intriguingly, we also observe significant changes in expression of genes and proteins encoding major Ca^2+^-handling proteins. Of note, the cardiac ryanodine receptor (RYR2) gene and protein is upregulated in KO-CM, as well as the cardiac sodium channel (SCN5A, SCN4B) while genes encoding the potassium channel (KCNE1) and the catalytic subunit alpha-2 of the sodium–potassium ATPase (ATP1A2) are downregulated. These changes on transcriptional and protein level could have the potential to give rise to functional changes influencing the Ca^2+^ homeostasis observed by CaT analysis of CRISPLD1-KO-CM. However, these assumptions need to be validated on functional level (e.g. by measuring action potential duration, SR Ca^2+^load, intracellular Ca^2+^/Na^+^ and K^+^ concentrations and/or—currents) in future studies.

We propose that the significant increase in CaT amplitude and the fast Ca^2+^ decay after CRISPLD1-KO might lead to a beneficial effect in human iPSC-CM in response to stress as a result of Ca^2+^ cycling blockade. Our data gained from toxin treatment and rescue experiments in WT-hiPSC-CM and CRISPLD-KO-hiPSC-CM support this hypothesis, as disturbed CaT after treatment with the Ca^2+^ blocking toxin helothermine were rescued in CRISPLD1-KO-hiPSC-CM. A beneficial effect of CRISPLD1 loss-of-function in hiPSC-CM is further supported by transcriptomics, proteomics and pathways analysis of CRISPLD1-KO- vs. WT-CM. However, the detailed molecular mechanism of CRISPD1-KO affecting Ca^2+^ cycling and the downstream effect on CM contractile performance need to be validated in future studies. Nonetheless, the progressive increase in CRISPLD1 expression during disease progression in AS patients points towards a functional role in the transition to HF, presumably as a negative regulator of Ca^2+^ cycling and thus, with the potential to augment CaT in the transition to HF to ameliorate cardiac contractility.

## Materials and methods

### Sampling of human myocardium

Human ventricular myocardium was obtained from biopsies of patients undergoing aortal valve implantation with morrow resection CH (*n* = 5, EF = 55% [58.4 ± 2.9%]) and mHF (*n* = 5, EF = 33% [33.5 ± 4.1%]). Detailed clinical parameters of patients are listed in suppl. Table 1. Freshly explanted donor hearts served as healthy control myocardium (NF, *n* = 4) and left ventricular myocardium of explanted hearts of four end-stage heart failure patients undergoing cardiac transplantation surgery as a result of ischemic or dilated cardiomyopathy were used for the analysis of the tHF stage. Directly after collection, myocardium samples were snap frozen in liquid nitrogen and stored at − 80 °C. The investigation conforms with the principles outlined in the Declaration of Helsinki. The study was approved by the institutional ethics committee, and all patients provided written informed consent for the use of cardiac tissue samples.

### Sampling of mouse myocardium

The investigation conforms to the Guide for the Care and Use of Laboratory Animals (NIH publication No. 85–23, revised 1996) and was performed in accordance with the ethical standards laid down in the Declaration of Helsinki 1964. Transverse aortic constriction (TAC) surgery was done using a minimally invasive approach as described previously [25]. Briefly, 8-week-old female FVBN mice were anesthetized using intraperitoneal injections of a mixture of xylazine and ketamine. A 27 gauge needle was tied against the aorta using a 5-0 non-absorbable suture. After aortic constriction, skin was closed and the mice were kept on a heating plate until recovering from anesthesia. Sham animals underwent the same procedure except banding of the transverse aorta. Mice were anesthetized with isoflurane and sacrificed by cervical dislocation. The body weight was determined before dissection. The heart was excised at the aorta and transferred into a petri dish filled with sterile saline. A 21 gauge blunt needle was used for retrograde perfusion with sterile saline to remove residual blood. The heart was weighted, atria and the right ventricle removed, and the left ventricle weighted again before it was snap frozen in liquid nitrogen. Mice echocardiography and morphometry data are shown in suppl. Fig. S1.

### Echocardiography

The mice were anesthetized using 1.5% isoflurane, and echocardiography was performed using a VS-VEVO 660/230 (Visualsonics, Toronto, Canada). 2D-guided M-mode images were recorded in the long-axis view at the left mid-ventricular level. The examiner was blinded towards group assignment.

### Direct differentiation of hiPSC into hiPSC-CM

Human iPSC were cultured in feeder-free culture conditions with StemFlex Medium (Thermo Fisher Scientific) on Matrigel (BD Bioscience)-coated plates. The experiments were performed with the hiPSC line ipWT1.3 (UMGi014-B.3) from a healthy donor, generated from dermal fibroblasts in feeder-free culture conditions using the integration-free episomal 4-in-1 CoMiP reprogramming plasmid (Addgene, catalog 63726) as described previously [12, 15]. Directed differentiation of hiPSC into mainly ventricular-like CM was performed by modulation of WNT signaling, as previously described [12, 15]. In brief, hiPSC with a confluence of 80–90% were treated with Cardio Differentiation Medium (RPMI 1640 with Glutamax and HEPES (Thermo Fisher Scientific), 0.5 mg human recombinant albumin (Sigma-Aldrich) and 0.2 mg/ml l-ascorbic acid 2-phosphate (Sigma-Aldrich) supplemented with 4 µM or 6 µM CHIR99021 (Merck Millipore) for 48 h. Subsequently, CHIR99021 was exchanged to 5 µM IWP2 (Merck Millipore) for further 48 h. First beating areas were observed around day 8 and medium was changed to Cardio Culture Medium (RPMI 1640 with Glutamax and HEPES + 2% B27 (Thermo Fisher Scientific), respectively. Metabolic selection was performed with Cardio Selection Medium (RPMI 1640 without glucose and without HEPES (Thermo Fisher Scientific), 0.5 mg/ml human recombinant albumin, 0.2 mg/ml l-ascorbic acid 2-phosphate, 4 mM lactate (Sigma-Aldrich) and 4 mM HEPES (Sigma-Aldrich) for 5 days. To achieve lower cell densities, differentiated cultures were digested on day 20 with 0.25% Trypsin/EDTA (Thermo Fisher Scientific). Afterwards, cardiomyocytes were cultured for another 7 days with Cardio Selection Medium before changing to Cardio Culture Medium for further maturation until day 60. Differentiation efficiency was examined by observation of contracting areas and flow cytometry. In this study, only differentiated cultures with > 90% contracting areas were used.

### Generation of KO-hiPSC clones by CRISPR/Cas9

Transfection of the wild-type hiPSC line ipWT1.3 with the all-in-one Cas9 and guide RNA (gRNA) expression plasmid (Sigma-Aldrich) targeting CRISPLD1 exon 7 was performed with the Human Stem Cell Nucleofector Kit 2 (Lonza: VPH-5022) and the Amaxa Nucleofector II Device (Lonza) according to manufacturer’s instructions. In brief, hiPSC with 80–90% confluency were pretreated with 2 µM TZV 1 h before transfection. For nucleofection, 2 × 10^6^ cells were resuspended in DNA–nucleofector mix with 2 µg CRISPR/Cas9 plasmid and nucleofected using program B-016. Subsequently, cells were replated in one well of a six-well plate containing StemFlex Medium (Thermo Fisher Scientific), 2 µM TZV and 1 × Pen/Strep. 24 h after transfection, plasmid- and GFP reporter-positive hiPSC were sorted by fluorescence activated cell sorting (FACS). Positive transfected cells were seeded at 1 cell per well on a 96-well plate containing MEF-conditioned MACS medium (Miltenyi Biotech), 1 × Pen/Strep, 2 µM TZV and 50 ng/µl bFGF. First colonies were visible after 10–18 days and medium was changed to MACS medium. HiPSC clones were expanded and analyzed at genomic level via sequencing. The QIAamp DNA Mini kit (Qiagen) was used for genomic DNA isolation. DNA products for sequencing were purified after agarose gel electrophoresis using the QIAquick Gel extraction kit (Qiagen). The PCR products were validated by sequencing (SeqLab, Goettingen). Successfully edited clones were sequenced at mRNA level for verification of posttranscriptional changes induced by nucleotide deletion using the SV Total RNA Isolation System (Promega) and RT2 First strand kit (Qiagen) for cDNA synthesis.

### Confocal calcium imaging

Three independent differentiation experiments of the CRISPLD1-KO and the corresponding isogenic WT hiPSC line ipWT1.3 were used for cytosolic calcium recordings. Cells were replated on matrigel-coated glass cover slips (three replicates/differentiation) in lower densities following 7 days of recovery. For recordings, hiPSC-CM at day 60–80 were incubated with 5 µM Rhod-2 AM fluorescent calcium indicator (Thermo Fisher Scientific) and 0.02% (w/v) Pluoronic F-127 (Thermo Fisher Scientific) in Tyrode`s solution (140 mM NaCl, 5.4 mM KCl, 1.8 mM CaCl_2_, 1 mM MgCl_2_, 10 mM HEPES, and 10 mM glucose (pH 7.4) for 30 min at RT. Recordings were obtained from paced cells in Tyrode’s solution only or in Tyrode’s solution supplemented with 5 µM helothermine at RT which were field stimulated at 0.25 Hz (18 V, 3 ms duration; MyoPacer ES, IonOptix) using a recording chamber with platinum electrodes. For acquisition, the LSM 710 confocal microscopy system (Carl Zeiss) with a 63 × /1.4 NA oil objective and Zen 2009 software was used and images were captured in line-scan mode (512 pixels, 45 μm, 1057.7 Hz, 20,000 cycles, pinhole 6 AU). Rhod-2 AM was excited at 561 nm and emission was detected at 566–646 nm. Post-acquisition analyses of changes in intracellular calcium were performed by plotting of mean signal intensity as a function of time using ImageJ (NIH), polynomial smoothing (6th order, 10 neighbors) of raw data using Excel, and final analysis of calcium transients using the Peak Analysis Tool in LabChart Pro 8 (ADInstruments) with the following settings: automatic recognition of resting membrane potential, TStart 15% of height away from resting membrane potential, TRise and TFall is defined between 0 and 100% of the peak height. Calcium transient (CaT) decay 50 was defined as the time from the maximum of the transient until 50% signal decay. CaT rise time was defined as the time from start to the maximum of the transient. For each analyzed cell, the individual data set represents a mean of 4 CaT recordings. In total, 181 WT cells and 183 KO cells were measured. Datasets were analyzed using Excel and R. Statistics were performed using two-tailed student’s *t*-test and one-way ANOVA with random effects. Data are presented as mean ± SD.

### Immunocytochemical staining

Human iPSC-CM were replated in lower density on matrigel-coated glass cover slips. After 7 days of recovery, cells were fixated with 4% PFA for 20 min at RT and subsequently blocked in 1% bovine serum albumin (BSA; Sigma-Aldrich) in phosphate buffered Saline (PBS) at 4 °C. The primary antibody was incubated overnight at 4 °C in 1% BSA supplemented with 0.1% Triton X-100 (Carl Roth) for cell permeabilization. Secondary antibody was incubated for 1 h at RT. Afterwards, Hoechst33342 was incubated for 10 min at RT before glass cover slips were mounted with Fluoromount-G (Thermo Fisher Scientific) on microscope slides. Image recording was performed using the LSM 710 confocal microscopy system. Antibody information is given in suppl. Table 3.

### RNA extraction

Human myocardium samples were homogenized using 5 mg/sample in 500 µl of qiazol (Qiagen). Samples were homogenized for 30 s using MICCRA D1 dispersing instrument at level F (35,000 min^−1^). Total RNA was extracted using miRNeasy blood and tissue kit (Qiagen) following manufacturers protocol. Human iPSC-CM pellets were snap frozen in liquid nitrogen. For total RNA isolation of cells and mice left ventricle, the SV Total RNA Isolation System (Promega) or the RNeasy Fibrous Tissue mini Kit (Qiagen) was used.

### Protein extraction and proteome analysis

iPSC-CM were washed and transferred with 1 ml 1 × PBS into an Eppendorf tube. Cells were centrifuged at 13,000 rpm for 1 min and resuspended in 50 µl RIPA buffer (Thermo Fisher Scientific). After incubation for 10 min, cells were centrifuged at 4 °C for 5 min at 5000 rpm and the supernatant was used for analysis. Protein concentration was determined using the BCA Assay as described in the manufacturer’s instructions (Thermo Fisher Scientific). The Generation, quantification and analysis of proteome data is detailed in the supplementary methods.

### Quantitative real-time PCR

200 ng of isolated RNA were used for first-strand cDNA synthesis (RT2 First strand Kit; Qiagen) according to the manufacturer`s instructions. Quantitative real-time PCR was performed with RT2 SYBR Green qPCR Mastermix (Qiagen) and the CFX96 Real-Time System Thermal Cycler (BioRad). The results were analyzed with the ddCT method in Excel and normalized to H18S and GAPDH, respectively. T test statistics were performed with GraphPad Prism 7. Primer information qPCR analyses are listed in suppl. Table 4.

### mRNA sequencing

mRNA-seq was performed at the Transcriptome and Genome Analysis Laboratory (TAL) core facility in Goettingen. Library preparation was conducted according to the instructions of the TruSeq RNA Sample Preparation v2 Kit from Illumina (Cat. No. RS-122-2002) using 1 µg total RNA as starting material. For accurate quantitation of cDNA libraries, fluorometric-based QuantiFluor™ dsDNA System from Promega (Mannheim, Germany) was used. The size of final cDNA libraries was determined using the DNA 1000 chip (280 bp) on the Bioanalyzer 2100 (Agilent). cDNA libraries were amplified and sequenced (SR; 1 × 50 bp; ca. 40 Mio reads per sample) using the cBot and HiSeq2000 (Illumina). Sequence images were transformed to bcl files with the software BaseCaller (Illumina), and then demultiplexed to fastq files with CASAVA v1.8.2. Quality checking was done via fastqc. Sequences were aligned to the genome reference sequence of *Homo sapiens* (GRCh38/hg38) or *Mus musculus* (GRCm38/mm10), respectively. The alignment was performed using the STAR alignment software (version 2.3.0e) 1 allowing for two mismatches within 50 bases. Subsequently, conversion of resulting SAM files to sorted BAM files, filtering of unique hits and counting were conducted with SAMtools (version 0.1.19) 2 and HTSeq (version 0.6.1p1) 3. Data were preprocessed and analyzed in the R/Bioconductor environment (www.bioconductor.org) using the DESeq2 package (version 1.8) 4. Specifically, the data were normalized and tested for differentially expressed genes based on a generalized linear model likelihood ratio test assuming negative binomial data distribution. Candidate genes were filtered to a minimum of log_2_FC > 1/− 1 and a false discovery rate–corrected *p *value < 0.05. Gene annotation was performed using Homo sapiens entries from Ensembl (www.ensembl.org) via the biomaRt package (version 2.18.0) 5.

### GO and pathway enrichment

Pathway analysis was conducted using significantly regulated genes (cut off log_2_FC > 1/− 1; adjusted *p *value < 0.05 and cut off log_2_FC > 0.5/− 0.5; adjusted *p *value < 0.05 in case of iPSC-CM) of the transcriptomes using Cytoscape and ClueGO [6, 41] and KEGG mapper—search and color pathway.

### Statistics

CaT analysis and animal data were analyzed and are presented using standard deviation (± SD). All other data were analyzed and are presented as mean standard error of the mean (± SEM) if not indicated otherwise. All data were statistically analyzed using Graph pad prism 7 or excel. Differences by unpaired student’s *t* test were considered significant when *p *value ≤ 0.05 (or FDR-corrected *p *value ≤ 0.05 for RNA-seq results, respectively). Not significant: (n.s.) *p *value > 0.05; *p *value ≤ 0.05 = *; *p *value ≤ 0.01 = **; *p *value ≤ 0.001 = ***; *p *value ≤ 0.0001 = ****.

### Study approval

The investigation conforms to the principles outlined in the Declaration of Helsinki. The use of patient biopsies was approved by the institutional ethics committee (approval number: 21/2/11). All patients provided written informed consent for the use of cardiac tissue samples. The use of hiPSC was approved by the institutional ethics committee (approval number: 10/9/15) and carried out in accordance with the approved guidelines. All mouse experiments (approval number: 13/1291) conform to the Guide for the Care and Use of Laboratory Animals (NIH publication No. 85–23, revised 1996) and were performed in accordance with the ethical standards laid down in the Declaration of Helsinki 1964.

## Electronic supplementary material

Below is the link to the electronic supplementary material.

Supplementary file1 (PDF 3241 kb)

Supplementary file8 (XLSX 388 kb)

Supplementary file9 (XLSX 656 kb)

Supplementary file10 (XLS 2557 kb)

Supplementary file11 (XLSX 209 kb)

Supplementary file12 (XLSX 22 kb)

Supplementary file13 (XLSX 31 kb)

Supplementary file14 (XLSX 1226 kb)
