# Supplementary material for: CRISPLD1: a novel conserved target in the transition to human heart failure
Source: Basic Res Cardiol. 2020 Mar 7;115(3):27. doi: 10.1007/s00395-020-0784-4 (PMC7060963; doi:10.1007/s00395-020-0784-4)
Supplement: Supplementary file 1 — Supplementary file1 (PDF 3241 kb) [file 395_2020_784_MOESM1_ESM.pdf]

CRISPLD1: – a novel conserved target in the transition to human heart failure

Authors: Sara Khadjeh[1, 2]*†, Vanessa Kernke[1, 2]†, Frederike Weber[1, 3], Lukas Cyganek[2, 3], Ramon O. Vidal[4, 5], Setare Torkieh[1], Katrin Streckfuss-Bömeke[1, 2], Dawid Lbik[1, 2], Malte Tiburcy[2, 6], Belal A. Mohamed[1, 2], Stefan Bonn[4,7, 8], Karl Toischer[1, 2]†, Gerd Hasenfuss[1, 2]†.

[1] Laboratory of Experimental Cardiology, Clinic for Cardiology and Pneumology, Heart Research Center, University Medical Center Goettingen, Goettingen, Germany

[2] German Centre for Cardiovascular Research (DZHK), partner site Goettingen, Germany

[3] Stem Cell Unit, Clinic for Cardiology and Pneumology, University Medical Center Goettingen, Goettingen, Germany

[4] German Center for Neurodegenerative Diseases (DZNE) Goettingen Site, Goettingen, Germany

[5] Scientific Genomics Platform, BIMSB, Max Delbrück Center for Molecular Medicine, Berlin, Germany

[6] Institute of Pharmacology and Toxicology, University Medical Center Goettingen, Goettingen, Germany

[7] Center for Molecular Neurobiology Hamburg, Institute of Medical Systems Biology, University Medical Center Hamburg-Eppendorf, Hamburg, Germany

[8] German Center for Neurodegenerative Diseases (DZNE), Tuebingen, Germany

† Equally contributing, *Corresponding authors

Address correspondence to: Sara Khadjeh, University Medical Center Göttingen, Clinic for Cardiology and Pneumology, Heart Research Center, Robert-Koch-Str. 42a, 37075 Göttingen, Germany. Phone: +49-(0)551-39 63633, E-Mail: sara.khadjeh@med.uni-goettingen.de

Keywords

Heart failure, compensated hypertrophy, calcium cycling, iPSC-CM

Supplementary Materials

Supplementary methods:

Expression profiling and identification of conserved candidates.

Generation, quantification and analysis of proteome data.

Fig. S1: Echocardiographic analysis of sham and TAC mice.

Fig. S2: Expression profiling of transcriptomes of pressure overload induced hypertrophy and heart failure human and mouse myocardium.

Fig. S3: Principle component analysis of RNA-seq data derived from human myocardium samples.

Fig. S4: Principle component analysis of RNA-seq data derived from mouse myocardium samples.

Fig. S5: Validation of HF marker gene expression in human myocardium samples by Real-Time quantitative polymerase chain reaction (qPCR).

Fig. S6: Principle component analysis of RNA-seq data derived from hiPSC-CM WT-control and CRISPLD1-KO.

Fig. S7: Adrenergic signaling in WT-control versus CRISPLD1-KO-CM and validation by qPCR.

Information. S8: Sequence information of CRISPLD1-iPSC-KO.

Table S1: AS clinical characteristics.

Table S2: Literature RNA-seq candidates.

Table S3: Antibodies used in this study.

Table S4: qPCR primer used in this study.

Supplementary data Excel files:

Data file S1: Human mRNA expression profiles

Data file S2: Mouse mRNA expression profiles

Data file S3: DE ctrl vs CRISPLD1KO

Data file S4: ClueGO analysis CRISPLD1-KO down

Data file S5: ClueGO analysis CRISPLD1-KO up

Data file S6: KEGG adrenergic signaling molecules

Data_file_S7: Proteome analysis CRISPLD1-KO

Supplementary Literature

Supplementary methods

Expression profiling and identification of conserved candidates. Differential expression (DE) was assessed for the CH/1wTAC and HF group compared to the control/sham group. Differential expression analysis was done using DESeq2. Initial DE analysis of the control transcriptomes vs pathology (cut offs: minimal counts > 10, log2FC >+/-1, adjusted p value < 0.05) resulted in a list of significant DEGs for each of the conditions vs. the control. Each gene that were significantly differentially expressed in at least one DE analysis was analyzed for expression in the other condition and the control comparing the normalized counts (see data file S2 and S3) using the following criteria:

If the sum of two pathology groups were higher than 20 counts the medians were calculated. Fold changes higher than 1.5 received negative or positive labels depending on the expression between the 3 conditions. Fold changes below 1.2 indicate no alteration of expression between conditions. Exclusive expression is given by expression higher than 50 counts in one case and less than 10 counts in the other two. Data sets of human and mouse genes were compared under consideration of the expression profiles and 25 genes were identified showing conserved changes in expression during disease progression in human and mouse.

Generation, quantification and analysis of proteome data. For generation of a peptide library, equal amount aliquots from each sample were pooled to a total amount of 80 µg, and separated into eight fractions using a reversed phase spin column (Pierce High pH Reversed-Phase Peptide Fractionation Kit, Thermo Fisher Scientific). All samples were spiked with a synthetic peptide standard used for retention time alignment (iRT Standard, Schlieren, Schweiz).

Protein digests were analyzed on a nanoflow chromatography system (Eksigent nanoLC425) hyphenated to a hybrid triple quadrupole-TOF mass spectrometer (TripleTOF 5600+) equipped with a Nanospray III ion source (Ionspray Voltage 2400 V, Interface Heater Temperature 150°C, Sheath Gas Setting 12) and controlled by Analyst TF 1.7.1 software build 1163 (all AB Sciex). In brief, peptides were dissolved in loading buffer (1% acetonitrile, 0.1% Trifluoroacetic acid in water) to a concentration of 0.3 µg/µl. For each analysis 1.5 µg of digested protein were enriched on a µPAC Trapping column (micro-Chip Based Seperations, Symmetry C18, Pharma Fluidics) and separated on an analytical RP-C18 column (50cm µPAC RP C18, PharmaFluidics) using a 60 min linear gradient of 5-35 % acetonitrile/0.1% formic acid (v:v) at 300 nl min^{-1}.

Qualitative LC/MS/MS analysis was performed using a Top20 data-dependent acquisition method with an MS survey scan of *m/z* 350–1250 accumulated for 350 ms at a resolution of 30,000 full width at half maximum (FWHM). MS/MS scans of *m/z* 180–1600 were accumulated for 100 ms at a resolution of 17,500 FWHM and a precursor isolation width of 0.7 FWHM, resulting in a total cycle time of 2.9 s. Precursors above a threshold MS intensity of 125 cps with charge states 2+, 3+, and 4+ were selected for MS/MS, the dynamic exclusion time was set to 30 s. MS/MS activation was achieved by CID using nitrogen as a collision gas and the manufacturer's default rolling collision energy settings. Two technical replicates per reversed phase fraction were analyzed to construct a spectral library.

For quantitative SWATH analysis, MS/MS data were acquired using 65 variable size windows [28] across the 400-1,050 *m/z* range. Fragments were produced using rolling collision energy settings for charge state 2+, and fragments acquired over an *m/z* range of 350–1400 for 40 ms per segment. Including a 100 ms survey scan this resulted in an overall cycle time of 2.75 s. Two replicate injections were acquired for each biological sample.

Protein identification was achieved using ProteinPilot Software version 5.0 build 4769 (AB Sciex) at "thorough" settings. A total of 844.673 MS/MS spectra from the combined qualitative analyses were searched against the UniProtKB human reference proteome (revision 04-2018, 93.661 entries) augmented with a set of 52 known common laboratory contaminants to identify 4769 proteins at a False Discovery Rate (FDR) of 1%.

Spectral library generation and SWATH peak extraction were achieved in PeakView Software version 2.1 build 11041 (AB Sciex) using the SWATH quantitation microApp version 2.0 build 2003. Following retention time correction using the iRT standard, peak areas were extracted using information from the MS/MS library at an FDR of 1% [15]. The resulting peak areas were then summed to peptide and finally protein area values, which were used for further statistical analysis.

Supplementary figures

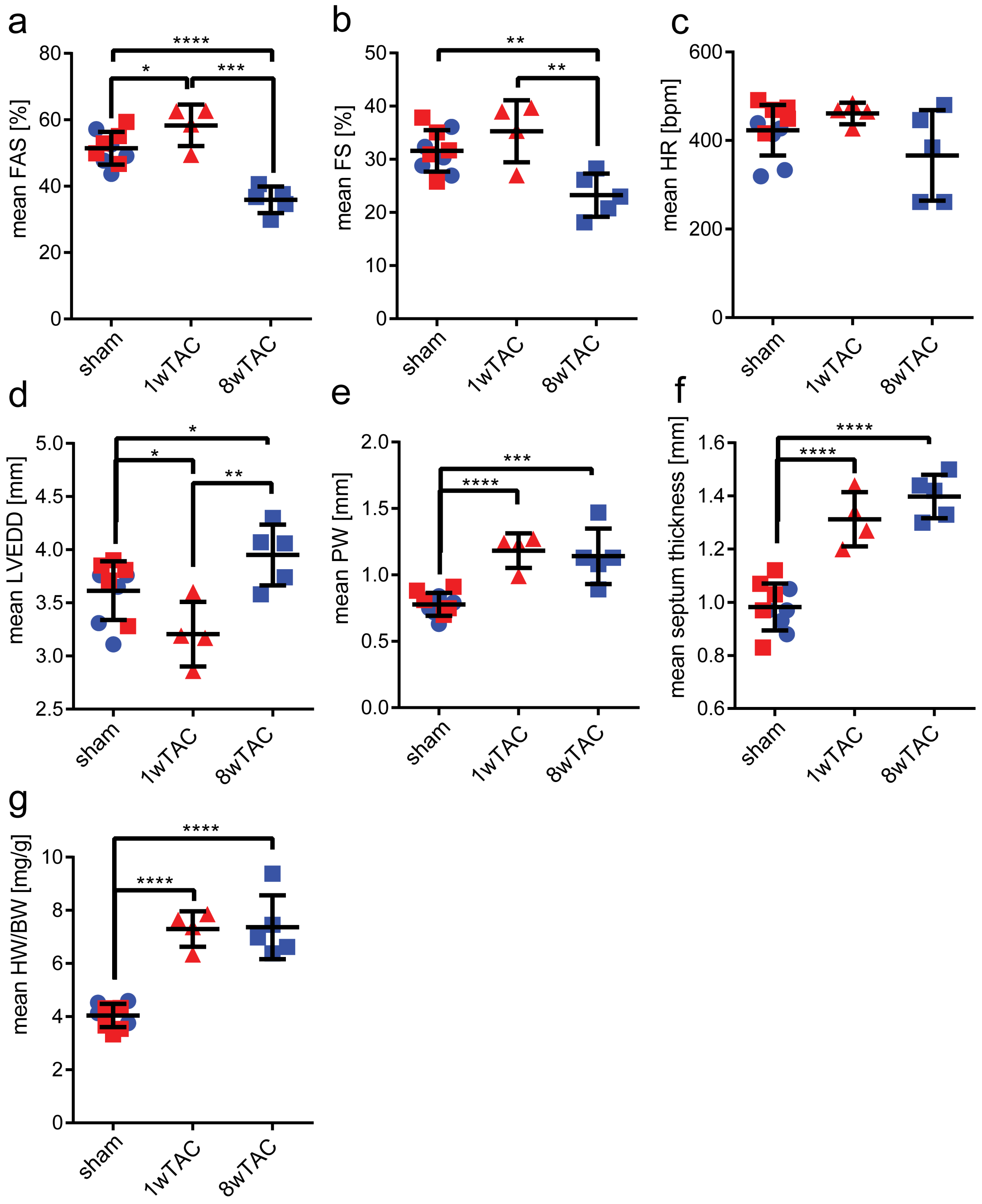

Supplementary figure S1: Echocardiographic analysis of sham and TAC mice. (a) Mean fractional area shortening (FAS), **(b)** mean fractional shortening (FS), **(c)** mean heart rate (HR), **(d)** mean left ventricular end diastolic diameter (LVEDD), **(e)** mean posterior wall thickness (PW), **(f)** mean septum thickness, **(g)** mean heart weight body weight ratio (mean HW/BW). Sham animals are highlighted in the corresponding color of treatment animals (blue 1wTAC, red 8wTAC). Error bars represent mean ± SD. 1wTAC = one week post transverse aortic constriction (TAC); 8wTAC = eight weeks post TAC. Mean of n= 4/5 per condition.

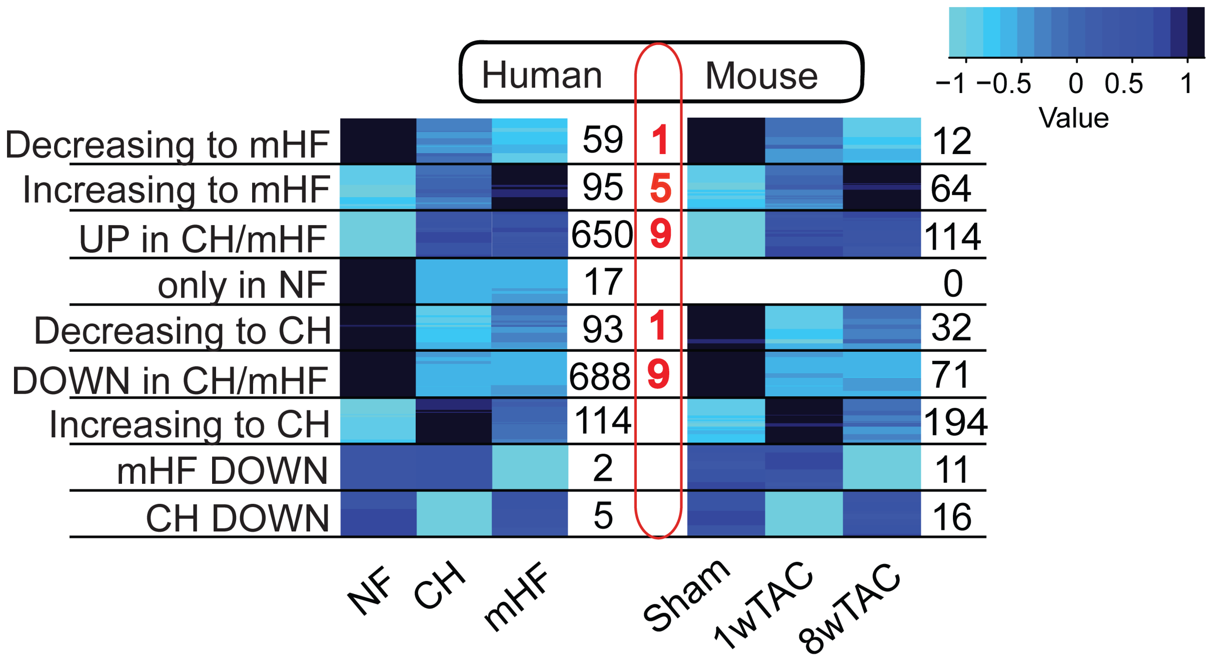

Supplementary figure S2: Expression profiling of transcriptomes of pressure overload induced hypertrophy and heart failure of human and mouse myocardium. Heatmap illustration of expression profiles of significantly differentially expressed genes in human and mouse transcriptomes during disease progression. The light blue to dark blue shading scale reflects the logFC (fold change) of median normalized counts. Black numbers next to the heatmap give the number of genes with the same expression profile. Genes expressed in the same way in human and mouse datasets according to disease progression (similar expression in the control, CH and mHF condition) are highlighted in red. NF = non-failing control, CH= compensated hypertrophy, mHF= moderate heart failure; 1wTAC= one week post transverse aortic constriction (TAC); 2wTAC= two weeks post TAC. n=4-5/group.

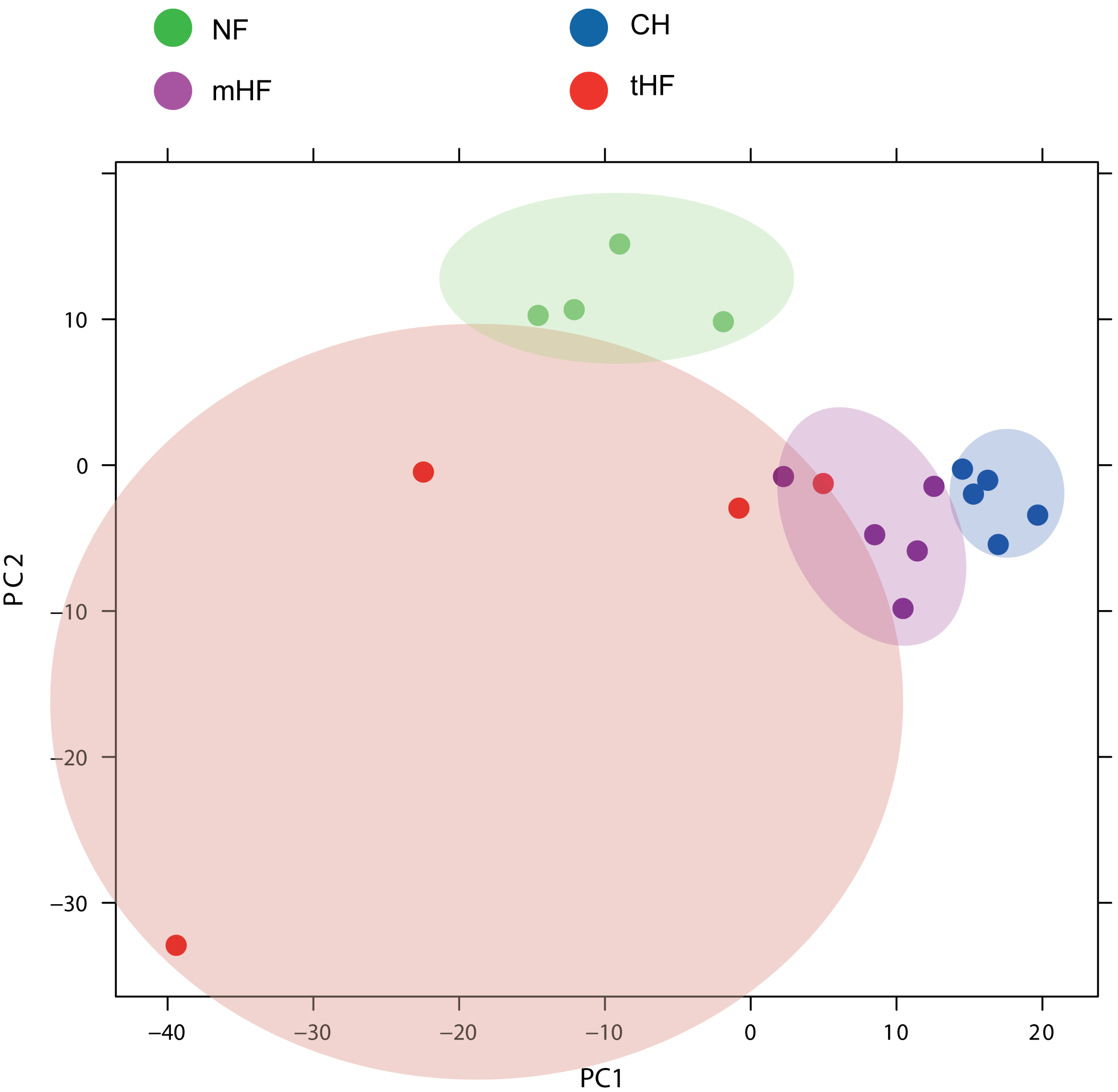

Supplementary figure S3: Principle component analysis of RNA-seq data derived from human myocardium samples. PCA visualizing the variation within principle component1 and 2. The analysis of left ventricular myocardium from non-failing control (NF, highlighted in green) samples, patients with compensated hypertrophy (CH, highlighted in blue), patients with moderate HF (mHF, highlighted in purple) and patients with terminally failing hearts (tHF, highlighted in red) as a result of dilated or ischemic cardiomyopathy shows a clear clustering of sample groups.

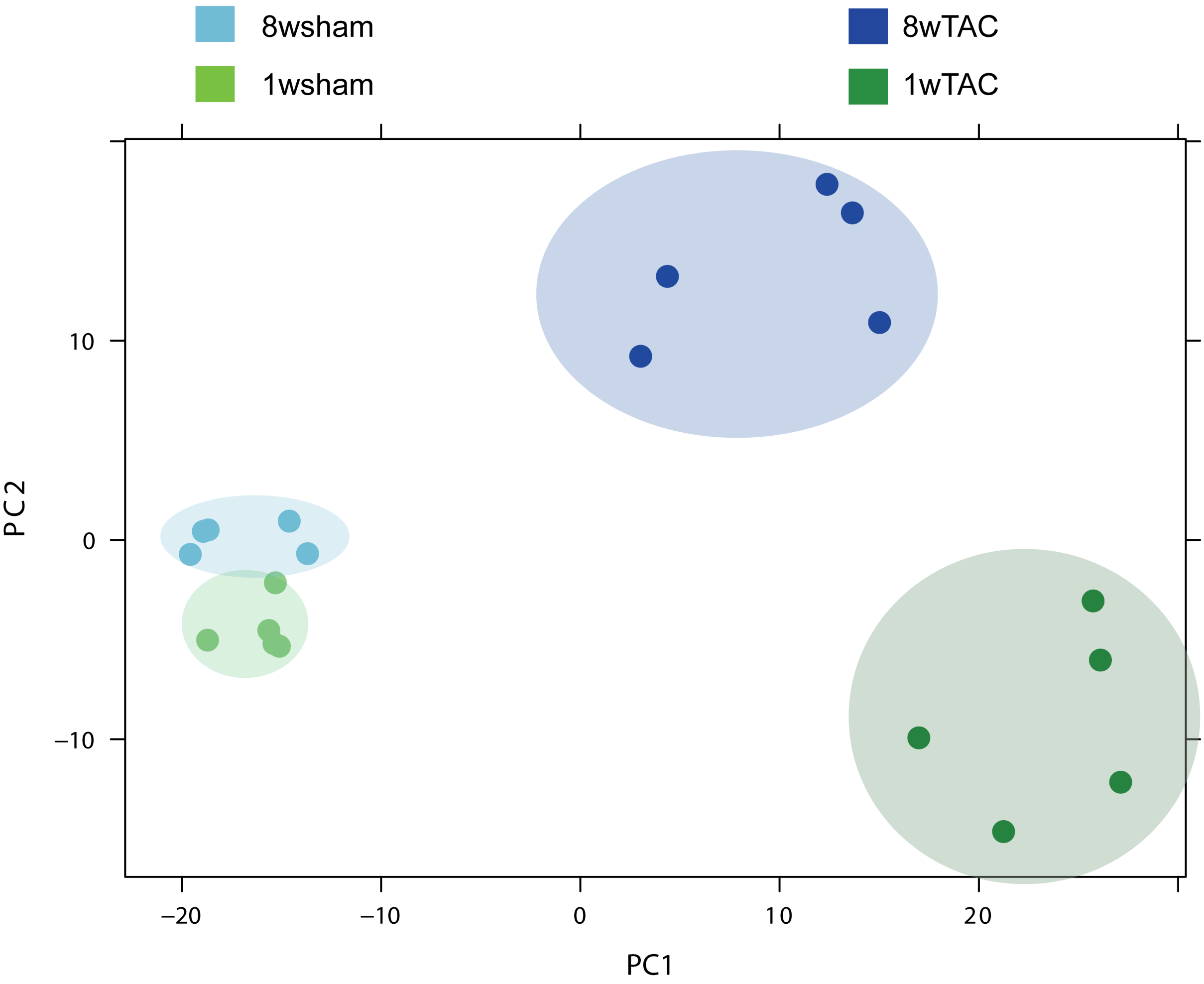

Supplementary figure S4: Principle component analysis of RNA-seq data derived from mouse myocardium samples. PCA visualizing the variation within principle component1 and 2. The analysis of left ventricular myocardium from sham control (1week post treatment = 1wsham, highlighted in light green, 8 weeks post treatment, highlighted in light blue) samples, mice one week post TAC (1wTAC, highlighted in green) and mice eight weeks post TAC (8wTAC, highlighted in blue) shows a clear clustering of sample groups.

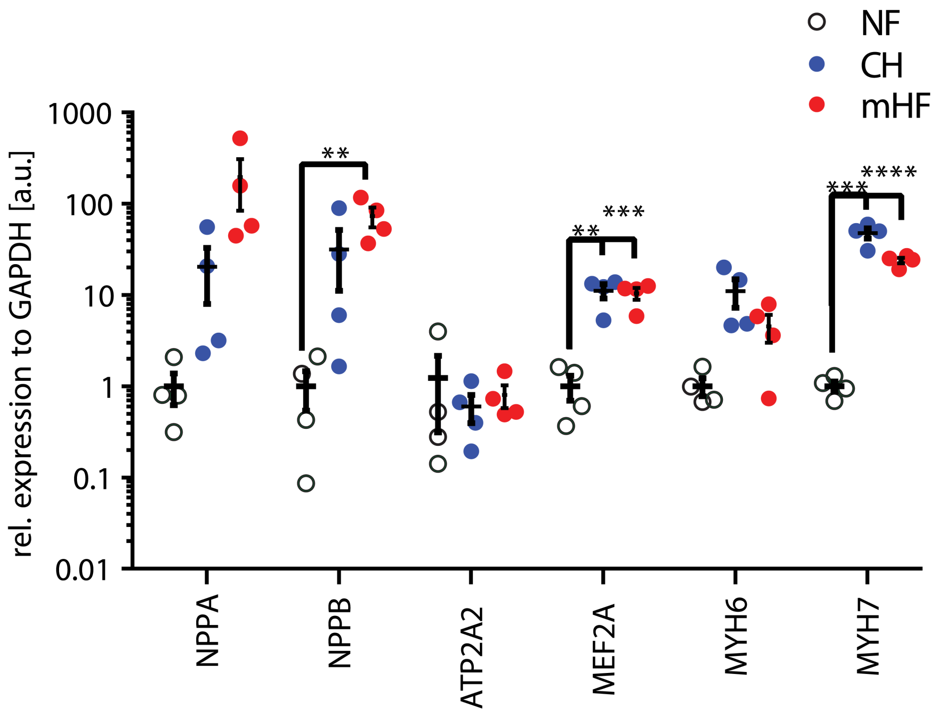

Supplementary figure S5: Validation of HF marker gene expression in human myocardium samples by Real Time quantitative polymerase chain reaction (qPCR). Shown is the gene expression of NPPA (Natriuretic Peptide A), NPPB (Natriuretic Peptide B), ATP2A2 (Sarcoplasmic/Endoplasmic Reticulum Calcium ATPase 2), MEF2A (Myocyte Enhancer Factor 2A, MYH6 (Myosin Heavy Chain 6) and MYH7 (Myosin Heavy Chain 7) relative to GAPDH. Replicate values are visualized by dots and represent n=4 cDNA libraries derived from human left ventricular myocardium samples of non-failing (NF, green), compensated hypertrophy (CH, blue) and moderate heart failure (mHF, red) groups. Error bars represent mean ± SEM. a.u. = arbitrary units. Each dot represents a mean value of n=3 qPCR technical replicates.

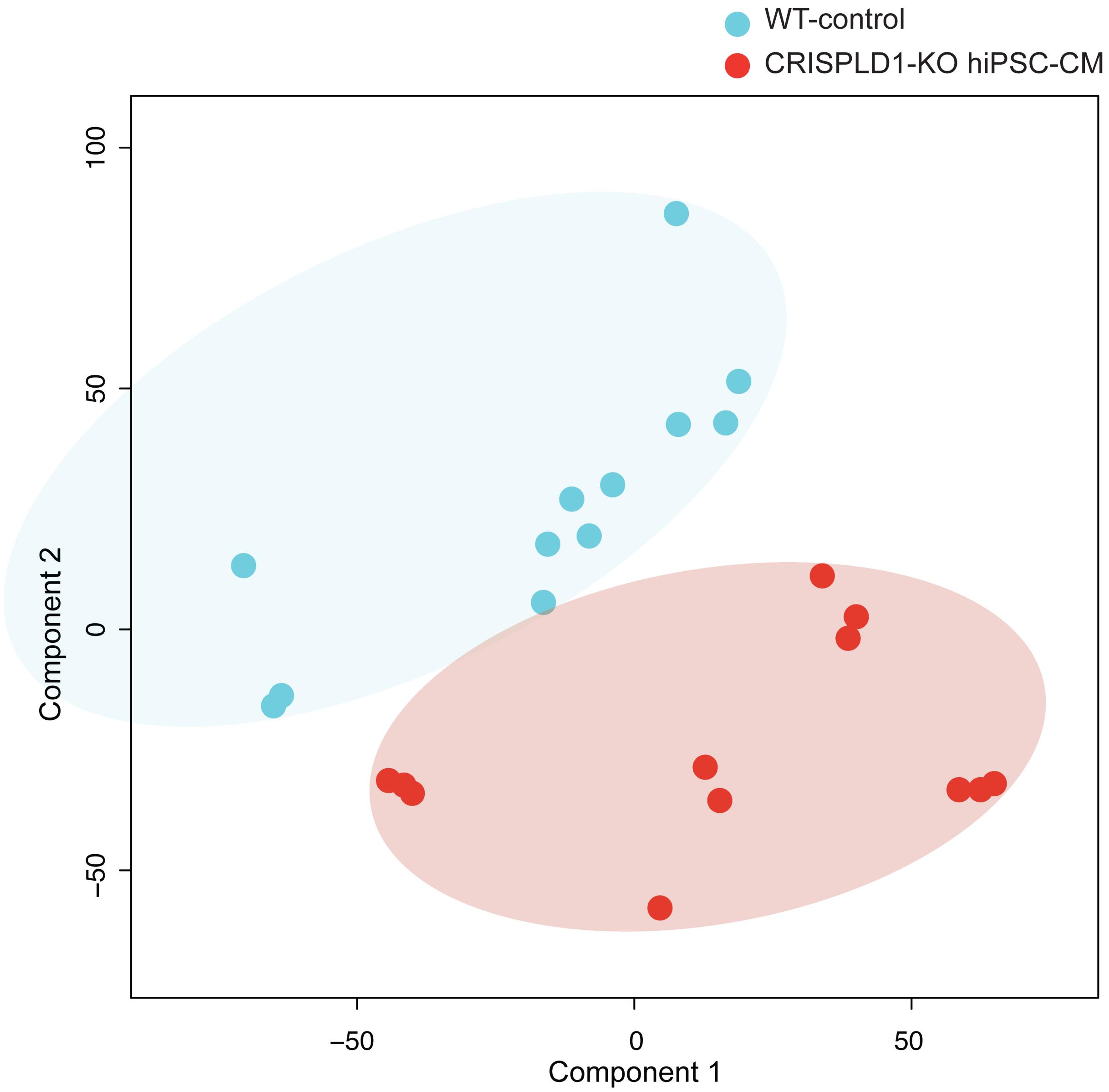

Supplementary figure S6: Principle component analysis of RNA-seq data derived from hiPSC-CM WT-control and CRISPLD1-KO. PCA visualizing the variation within principle component1 and 2. The analysis of hiPSC-CM-WT-control (highlighted in light blue) and CRISPLD1-KO-CM (highlighted in red) replicates shows a clear clustering of sample groups. Each data point represents a technical replicate of n= 4 independent differentiations per group. hipSC-CM = human induced pluripotent stem cell derived cardiomyocytes; CRISPLD1-KO-CM = CRISPLD1 knock out cardiomyocytes.

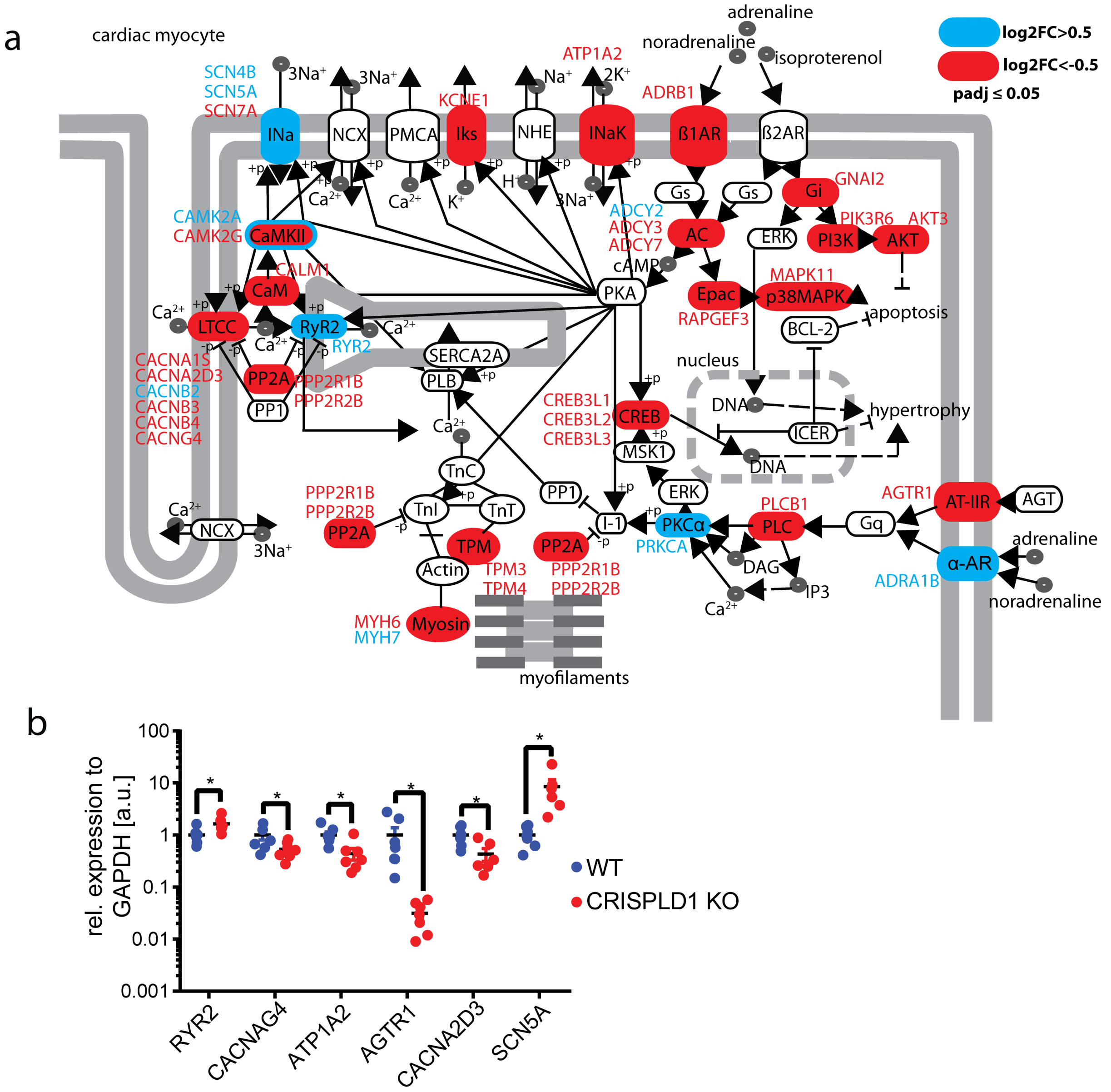

Supplementary figure S7: Adrenergic signaling in WT-control versus CRISPLD1-KO-CM and validation by qPCR. (a) Visualization of DEGs associated to adrenergic signaling in cardiomyocytes (pathway map adapted from KEGG entry hsa04261). Down regulated genes are highlighted in red, up regulated genes are in blue. Gene names are given next to the encoded Molecule. Most genes are downregulated in response to CRISPLD1-KO (see suppl. data S7 for normalized counts) like ADRB1 (adrenoreceptor beta 1), AGTR1 (angiotensin II receptor type 1) the L-type calcium channel namely CACNA2D3, CACNB3, CACNB4 and CACNG4, KCNE1 (potassium voltage-gated channel subfamily E regulatory subunit 1) and ATP1A2 (catalytic subunit alpha 2 of the Na+/K+ -ATPase). Genes encoding calmodulin 1 (CALM1), calcium/calmodulin dependent protein kinase II gamma (CAMK2G), protein phosphatase 2 (PPP2R1B, PPP2R2B), myosin heavy chain 6, tropomyosin (TPM3, TPM4), prohypertophic cAMP responsive element binding protein (CREB3L1, CREB3L2, CREB3L3), phospholipase C beta 1 (PLCB1), adenylate cyclase (ADCY3, ADCY7), Rap guanine nucleotide exchange factor 3 (RAPGEF3), mitogen-activated protein kinase 11 (MAPK11) and G

protein subunit alpha i2 (GNAI2), phosphoinositide-3-kinase regulatory subunit 6 (PIK3R6), AKT serine/threonine kinase 3 (AKT3) are also downregulated. SCN5A (α-subunit of the Na+ channel) SCN4B (sodium channel beta subunit) and RYR2 (cardiac ryanodine receptor) are upregulated as well as myosin heavy chain 7 (MYH7), protein kinase C alpha (PRKCA) and ADRAB1 (adrenoceptor alpha 1B). **(b)** qPCR of representative DEGs. Shown is the gene expression relative to GAPDH. Replicate values are visualized by dots representing n=6-7 cDNA libraries derived from independent differentiations of WT-control- (green) and CRISPLD1-KO-CM (red). Each dot represents a mean value of n=3 qPCR technical replicates. Error bars represent mean ± SEM. a.u. = arbitrary units.

Supplementary information S8: Sequence information of CRISPLD1-iPSC-KO.
CRISPLD1-iPSC-KO-DNA, mRNA and protein sequence after CRISPR/Cas9 mediated deletion.

ipWT1.3 CRISPLD1 iPSC KO genomic sequence

Deletion: 34 bp

```
CTCTATAATAAATTATATGAATGTCTTAAATCTATACGCCAAAATAATTA
CTTAGAATGTTCATATATAGTGATGTTTCACAATGTTTTATAAATTATGC
CATTAGACTACTTTTTAATAGAGCGTATCATCTCTTAAAGGGGAAACTGG
TGGGGCCATGCCCCTTACAAACATGGGCGGCCCTGTTCTGCTTGCCCACC
TAGTTTTGGAGGGGGCTGTAGAGAAAATCTGTGCTACAAAGGTAAGTGCT
ATTGTGTTGTGGTATTCATGTTGATTTATATTTTAATCCAGCTCCTGCAT
TACTTATGTTTAAAAAAGCCAGAAGTGCACTTAATGATCACACGAAAAGT
ATCTAGAACTCTATTTCAAATATTTTGAAGAAATCTTTTACAAATATTTA
TATTACTAATAAGTTTGTTATATGAAATTTTTAAAGTTCCATGGTTATTT
CAGGTAACTATTTCTGATTCTACTTTGAAATTACCCCAGAATGACAAGTA
TATTTATATTCTTAAAAAGAGTCATCTTGATGTATGGTTGACTAGAGTTA
TTTCCATAACTGTCATCTTTTTTTAAAGGATTTAGCAATTTAACACAATT
TATTTTTAGCGCTTTAAACATTTTTAACAAAATACCCCTTTGTGTATCTT
ATTGAACCATGCTCAATGAATTATGTTGATTTTTCACTTCTCACTGCACA
CTATTCAATTTGTCATGCTAAAATAATAAGAAAAGTATAATAGTAACATA
AAACTACTATAAAAGTAGTACTGTCAAATGCATTTGATATCTTAAAAAGT
AAATATGGTGTCCTGTTTATAGCACATAAAGATAAACTGTTATGACAAAG
CTTTGTGAAAAGTCATGCACTTGTGCATTCAAGTGAACATTTTTTACTGC
CTTGTACTTTTTTCTTGATGACTAAAAGAGGAATAATGTAGACGAAGTGT
ATTAGGAAATATTTGAACAATTGCAGCCCTTTTTTAGTGCTCATAATGAC
ACGTGTCTTTTTTTCCCTTAATGATGCATTAACTAAAATGAGTAGACAAG
AAGTGCCCATATATGTCCCTCTGTTGTGTAAGCCATTTTAAAATTCCTAG
AAGCGTTTGCAGCATTTTTTCCTTGTGTGGGTGTTTTTCATATTCTCTGT
GTCCAAACCTCCAACCTACCTGCCTCTTCCCCGGTGACCATTATCTTTTA
CTTAAAGCTGACTTTCTTGTGACTTCATACTTTTTTTTTAGGTTCTGAGT
TCTTGGGCATGCATCAAAATTATACACCTCTCCAAAATGTGCCATTGTCA
AGCCTTTGATAAAGGCCAGTCTGGAAATAAACATTAAATTCAAAGTTTGA
AATTACTCTCCATACTCAAGTTTAAATTTCTGCATATTGAATTTTCTTCT
AAAGGAAAAAAACTCTATGGACATGGATTTTGTCATAAATAATAAGGGCA
TGTAAATCCTTCTTTAGGCCACATTTCCATTTGGGAGAAGTCTAAATGTG
ATCCTCCTGAAAATATTAAGAATATGATTTTAATTGAAAACAAATTTGAT
TTTGCTAAGCCATGACCTTATTAGATCACTGTGTAGATTTCATGGTGTCA
TTATGAAAATCAAACAATGATATGTTGAAATATAAGCAAAGTTTAGAAGT
GGCTTCATTGAATATTAGTCGAACATTAACATAAATGAATCAAGGACTTA
AAGGCTTATGTGTGTTCATTTATTCCACTGCAGTACCTAAGATCACCACC
TTAAGCCAAAACCTGTGCAGAAAAACAGTACTGATTTTTCTAGTTCTAAA
TGTTAAATAGGAGTATTAAAGTATATTATTATTTGATAATTCATGCACAT
GTAAAATAGGGTTAATATTTCCTAAATCTGCTTTCTCTAACAGAAGGGTC
AGACAGGTATTATCCCCCTCGAGAAGAGGAAACAAATGAAATAGAAC---
-------------------------------GACAAGATCAGATGATAGT
AGCAGAAATGAAGTCATAAGCGCACAGCAAATGTGTAAGACCTCCATATT
ACTTATAAAAATTTTCAAAGTACATAAATGGTATATCCATCAAGATTTTA
TTAATTTGAACATTTTTAGAATGGAAAAAATTTTGTCATTAGGCTATATA
TAATGAACTACAACTGCTTTTATATTATTTAAGTTATTTAGGTATTTTTT
```

B3 mRNA Validation -> Exon 6 to 8 is spliced out (303 bp)

```
  1 ATGAAGTGTA CCGCGCGGGA GTGGCTCAGA GTAACCACAG TGCTGTTCAT
 51 GGCTAGAGCA ATTCCAGCCA TGGTGGTTCC CAATGCCACT TTATTGGAGA
101 AACTTTTGGA AAAATACATG GATGAGGATG GTGAGTGGTG GATAGCCAAA
151 CAACGAGGGA AAAGGGCCAT CACAGACAAT GACATGCAGA GTATTTTGGA
201 CCTTCATAAT AAATTACGAA GTCAGGTGTA TCCAACAGCC TCTAATATGG
251 AGTATATGAC ATGGGATGTA GAGCTGGAAA GATCTGCAGA ATCCTGGGCT
301 GAAAGTTGCT TGTGGGAACA TGGACCTGCA AGCTTGCTTC CATCAATTGG
351 ACAGAATTTG GGAGCACACT GGGGAAGATA TAGGCCCCCG ACGTTTCATG
401 TACAATCGTG GTATGATGAA GTGAAAGACT TTAGCTACCC ATATGAACAT
451 GAATGCAACC CATATTGTCC ATTCAGGTGT TCTGGCCCTG TATGTACACA
501 TTATACACAG GTCGTGTGGG CAACTAGTAA CAGAATCGGT TGTGCCATTA
551 ATTTGTGTCA TAACATGAAC ATCTGGGGGC AGATATGGCC CAAAGCTGTC
601 TACCTGGTGT GCAATTACTC CCCAAA---- ---------- ----------
651 ---------- ---------- ---------- ---------- ----------
701 ---------- ---------- ---------- ---------- ----------
751 ---------- ---------- ---------- ---------- ----------
801 ---------- ---------- ---------- ---------- ----------
```

```
 851 ---------- ---------- ---------- ---------- ----------
 901 ---------- ---------- ---------G TACGAATGTC CTGCTGGCTG
 951 TTTGGATAGT AAAGCTAAAG TTATTGGCAG TGTACATTAT GAAATGCAAT
1001 CCAGCATCTG TAGAGCTGCA ATTCATTATG GTATAATAGA CAATGATGGT
1051 GGCTGGGTAG ATATCACTAG ACAAGGAAGA AAGCATTATT TCATCAAGTC
1101 CAATAGAAAT GGTATTCAAA CAATTGGCAA ATATCAGTCT GCTAATTCCT
1151 TCACAGTCTC TAAAGTAACA GTTCAGGCTG TGACTTGTGA AACAACTGTG
1201 GAACAGCTCT GTCCATTTCA TAAGCCTGCT TCACATTGCC CAAGAGTATA
1251 CTGTCCTCGT AACTGTATGC AAGCAAATCC ACATTATGCT CGTGTAATTG
1301 GAACTCGAGT TTATTCTGAT CTGTCCAGTA TCTGCAGAGC AGCAGTACAT
1351 GCTGGAGTGG TTCGAAATCA CGGTGGTTAT GTTGATGTAA TGCCTGTGGA
1401 CAAAAGAAAG ACCTACATTG CTTCTTTTCA GAATGGAATC TTCTCAGAAA
1451 GTTTACAGAA TCCTCCAGGA GGAAAGGCAT TCAGAGTGTT TGCTGTTGTG
1501 TGA
```

Protein Sequence

CRISPLD1 Protein Isoform 1 WT
(Domain annotations after ensembl entry ENSP00000262207.4
(https://www.ensembl.org/Homo_sapiens/Transcript/ProteinSummary?db=core;g=ENSG00000124490;r=6:49692358-49713590;t=ENST00000339139) & InterPro database: https://www.ebi.ac.uk/interpro/protein/Q9H336)

MW = 56888 dalton
Deletion: position 210 - 310
CAP-Domain = 60 - 214
Allergen V5/Tpx-1-related, conserved site = 201 - 212
LCCL domain1 = 289 - 384
LCCL domain2 = 390 - 487

CRISPLD1 Protein Isoform 1 (ENSP00000262207.4)

```
MKCTAREWLRVTTVLFMARAIPAMVVPNATLLEKLLEKYMDEDGEWWIAK 50
QRGKRAITDNDMQSILDLHNKLRSQVYPTASNMEYMTWDVELERSAESWA 100
ESCLWEHGPASLLPSIGQNLGAHWGRYRPPTFHVQSWYDEVKDFSYPYEH 150
ECNPYCPFRCSGPVCTHYTQVVWATSNRIGCAINLCHNMNIWGQIWPKAV 200
YLVCNYSPKGNWWGHAPYKHGRPCSACPPSFGGGCRENLCYKEGSDRYYP 250
PREEETNEIERQQSQVHDTHVRTRSDDSSRNEVISAQQMSQIVSCEVRLR 300
DQCKGTTCNRYECPAGCLDSKAKVIGSVHYEMQSSICRAAIHYGIIDNDG 350
GWVDITRQGRKHYFIKSNRNGIQTIGKYQSANSFTVSKVTVQAVTCETTV 400
EQLCPFHKPASHCPRVYCPRNCMQANPHYARVIGTRVYSDLSSICRAAVH 450
AGVVRNHGGYVDVMPVDKRKTYIASFQNGIFSESLQNPPGGKAFRVFAVV 500
```

CRISPLD1 Protein Isoform 2 (ENSP00000429746.1)

```
MNMNATHIVHSGVLALYVHIIHRGNWWGHAPYKHGRPCSACPPSFGGGCR 50
ENLCYKEGSDRYYPPREEETNEIERQQSQVHDTHVRTRSDDSSRNEVISA 100
QQMSQIVSCEVRLRDQCKGTTCNRYECPAGCLDSKAKVIGSVHYEMQSSI 150
CRAAIHYGIIDNDGGWVDITRQGRKHYFIKSNRNGIQTIGKYQSANSFTV 200
SKVTVQAVTCETTVEQLCPFHKPASHCPRVYCPRNCMQANPHYARVIGTR 250
VYSDLSSICRAAVHAGVVRNHGGYVDVMPVDKRKTYIASFQNGIFSESLQ 300
NPPGGKAFRVFAVV*
```

CRISPLD1 Protein Isoform 3 (ENSP00000430105.1)

```
MNIWGQIWPKAVYLVCNYSPKGNWWGHAPYKHGRPCSACPPSFGGGCREN 50
LCYKEGSDRYYPPREEETNEIERQQSQVHDTHVRTRSDDSSRNEVISAQQ 100
MSQIVSCEVRLRDQCKGTTCNRYECPAGCLDSKAKVIGSVHYEMQSSICR 150
AAIHYGIIDNDGGWVDITRQGRKHYFIKSNRNGIQTIGKYQSANSFTVSK 200
VTVQAVTCETTVEQLCPFHKPASHCPRVYCPRNCMQANPHYARVIGTRVY 250
SDLSSICRAAVHAGVVRNHGGYVDVMPVDKRKTYIASFQNGIFSESLQNP 300
PGGKAFRVFAVV*
```

Protein Sequence iPSC KO clone (Deletion of 101 AA)

```
MKCTAREWLRVTTVLFMARAIPAMVVPNATLLEKLLEKYMDEDGEWWIAK 50
QRGKRAITDNDMQSILDLHNKLRSQVYPTASNMEYMTWDVELERSAESWA 100
ESCLWEHGPASLLPSIGQNLGAHWGRYRPPTFHVQSWYDEVKDFSYPYEH 150
ECNPYCPFRCSGPVCTHYTQVVWATSNRIGCAINLCHNMNIWGQIWPKAV 200
YLVCNYSPKYECPAGCLDSKAKVIGSVHYEMQSSICRAAIHYGIIDNDGG 250
WVDITRQGRKHYFIKSNRNGIQTIGKYQSANSFTVSKVTVQAVTCETTVE 300
QLCPFHKPASHCPRVYCPRNCMQANPHYARVIGTRVYSDLSSICRAAVHA 350
GVVRNHGGYVDVMPVDKRKTYIASFQNGIFSESLQNPPGGKAFRVFAVV  399
```

Supplementary table 1: Clinical characteristics of aortic stenosis patients. Given are clinical parameters and medications. EF = ejection fraction; LVEDD = left ventricular end diastolic diameter; PWTh = posterior wall thickness; AV area = aortic valve area; Vmax= maximum jet velocity; P mean = pressure gradient.

**Group**	EF >55% (compensated hypertrophy)					EF<55% (moderate heart failure)				
patient	1	2	3	4	5	1	2	3	4	5
sex	m	m	f	m	m	m	m	f	m	m
age	71	73	57	77	74	64	58	48	69	52
EF [%]	55	60	60	53	55	20	30	30	45	33
Septum [mm]	n.n.	14	12	15	17	13	16	11	14	14
LVEDD [mm]	n.n.	42	38	49	44	54	61	49	51	47
PWTh [mm]	n.n.	13	12	16	15	13	17	11	12	12
calculated AV area [cm²]	n.n.	0.4	0.95	0.9	n.n.	0.6	0.95	n.n.	0.35	0.7
Vmax (m/sec)	4.2	4.2	3.7	4	4.8	4.2	4	4.01	4.06	4.2
P mean [mmHg]	n.n.	64	39	37	n.n.	38	34.3	35	46	n.n.
Aortic regurgitation	1	0	0	1	1	0	0	0	1	0
Mitral valve regurgitation	0	0	0	2	0	2	2	1	0	1
Heart rhythm	SR	SR	SR	SR	SR	SR	SR	SR	SR	SR
Coronary artery disease	3	2	2	2	2	3	0	0	3	0
Diabetes	0	0	0	1	1	1	1	0	1	0
ACE-inhibitor AT1-blocker	1	1	0	1	1	1	1	0	1	0
ß-blocker	0	1	1	1	1	0	1	1	1	0
catecholamines	0	0	0	0	0	0	0	0	0	0
ASS	1	1	1	1	1	1	1	1	1	0
Statine	0	1	1	0	1	1	1	0	1	0
antiarhythmic medication	0	0	0	0	0	0	0	0	0	0

Supplementary table 2: Literature research of conserved candidates identified by RNA-seq. Given is the regulation in the transition to failure, the gene symbol and name, whether it is found to be described in compensated hypertrophy (CH) and heart failure (HF) in the existing literature and the related publication.

Regulation	symbol	name	described in CH or HF	publication
progressivly upregulated	Crispld1	cysteine-rich secretory protein LCCL domain containing 1 [Source:MGI Symbol;Acc:MGI:1934666]	No	
	Fmod	fibromodulin [Source:MGI Symbol;Acc:MGI:1328364]	Upregulated in HF and implicated in fibrosis after TAC	e.g.: [2, 21, 26]
	Kif1a	kinesin family member 1A [Source:MGI Symbol;Acc:MGI:108391]	No- but other kinesins in the motorized traffic of cardiac ion channels.	[1]
	Nppa	natriuretic peptide type A [Source:MGI Symbol;Acc:MGI:97367]	encodes ANP	e.g.: [9, 17, 22]
	Comp	cartilage oligomeric matrix protein [Source:MGI Symbol;Acc:MGI:88469]	Icreased in patients with coronary artery disease, implicated in fibrosis after TAC	[21, 23]
progressivly downregulated	Aqp4	aquaporin 4 [Source:MGI Symbol;Acc:MGI:107387]	AQP4 knockout mice are prone to cardiac failure and arrhythmias through abnormal expressions of calcium handling proteins and exacerbate pro-inflammatory factors in the myocardium	[7]
down regulated CH	SFRP5	secreted frizzled-related protein 5 [ENSG00000120057]	AngiotensinII increases secreted frizzled related protein 5 (sFRP5) expression through AT1receptor/Rho/ROCK1/JNK signallling in cardiomyocytes. MiR-125b regulates SFRP5 expression activating cardiac fibroblasts.	[5, 12]
upregulated CH & mHF	LRRN2	leucine rich repeat protein 2, neuronal [Source:MGI Symbol;Acc:MGI:106037]	no – but the related Lrrc10 is required for early heart development and function in zebrafish and is a cardiac-specific factor regulating EC coupling in mice	[8, 13]
	RTN4	reticulon 4 [Source:MGI Symbol;Acc:MGI:1915835]	Identification of Nogo (RTN4) as novel indicator of HF	[6]
	FZD1	frizzled homolog 1 (Drosophila) [Source:MGI Symbol;Acc:MGI:1196625]	Receptor for Wnt proteins. Wnt-Fzd signalling plays an important role in cardiac function and disease	[10, 14]
	TTC9	tetratricopeptide repeat domain 9 [Source:MGI Symbol;Acc:MGI:1916730]	no	
	ST8SIA2	ST8 alpha-N-acetyl-neuraminide alpha-2,8-sialyltransferase 2 [Source:MGI Symbol;Acc:MGI:106020]	Involved in regulated and aberrant glycosylation that modulates cardiac electrical signaling	[18]
	COL8A2	collagen, type VIII, alpha 2 [Source:MGI Symbol;Acc:MGI:88464]	Identified in end-stage HF patients with dilated cardiomyopathy	[29]-
	FAM198	family with sequence similarity 198,	Was found to be associated with hypertrophy	[16]

	B	member B [Source:MGI Symbol;Acc:MGI:1915909]	and heart-faiure in rats.	
	SEMA3 D	sema domain, immunoglobulin domain (Ig), short basic domain, secreted, (semaphorin) 3D [Source:MGI Symbol;Acc:MGI:1860118]	plays a role in cardiomyocyte development.	[20]
	RUNX2	runt related transcription factor 2 [Source:MGI Symbol;Acc:MGI:99829]	Transcription factor which plays a role in heart development. central transcriptional regulator of osteoblast cell fate. Represses calcium deposition that causes progressive aortic valve disease.	[11]
downregulated CH & mHF	Gnmt	glycine N-methyltransferase [Source:MGI Symbol;Acc:MGI:1202304]		no
	Fitm1	fat storage-inducing transmembrane protein 1 [Source:MGI Symbol;Acc:MGI:1915930]	Mice lacking Fitm1 show improved profiles upon pressure overload induced HF	[19]
	Eci1	enoyl-Coenzyme A delta isomerase 1 [Source:MGI Symbol;Acc:MGI:94871]	encodes key mitochondrial enzyme involved in beta-oxidation of unsaturated fatty acids.	[25]
	Il15	interleukin 15 [Source:MGI Symbol;Acc:MGI:103014]	Was found as novel genetic variants contributing to left ventricular hypertrophy	[3]
	Gpt	glutamic pyruvic transaminase, soluble [Source:MGI Symbol;Acc:MGI:95802]	NO- plays a key role in intermediary metabolism of glucose and aminoacids	NCBI summ ary Gene ID: 2875
	Ech1	enoyl coenzyme A hydratase 1, peroxisomal [Source:MGI Symbol;Acc:MGI:1858208]	Protein is reduced in mouse CH and HF. Encoded protein localizes to peroxisomes. Functions in the auxiliary step of the fatty acid beta-oxidation pathway.	[4]
	Hsbp1l1	heat shock factor binding protein 1-like 1 [Source:MGI Symbol;Acc:MGI:1913505]	No	
	Selenbp1	selenium binding protein 1 [Source:MGI Symbol;Acc:MGI:96825]	Association mapping of cardiac remodeling and gene expression identified Selenbp1 cQTL associated with ISO-induced cardiac remodeling traits	[24]
	Slc36a2	solute carrier family 36 (proton/amino acid symporter), member 2 [Source:MGI Symbol;Acc:MGI:1891430]	Intergenic SNP found in Genome-Wide Association Study of a Heart Failure-Related Metabolomic Profile among African Americans in the Atherosclerosis Risk in Communities (ARIC) Study	[27]

Supplementary table 3: List of antibodies used in this study. Given is the antibody name, species and host species, dilution used in this study, the company and the related ordernumber.

**Antibody Name**	**Species**	**Host**	**Dilution (if tested)**	**Company**	**Order number**
α-actinin	several	mouse	1:1000 (IF)	Sigma	A7811
Ryr2	human	rabbit	1:500 (IF)	Sigma	HPA020028
cTNT	several	mouse	1:200 (IF)	Thermo	MA5-12960
Connexin43	several	rabbit	1:1000 (IF)	abcam	ab11370
MLC2V	human, mouse, rat, zebrafish	rabbit	1:200 (IF)	proteintech	10906-1-AP
goat anti-mouse AF555			1:1000	abcam	ab150114
goat anti-rabbit AF488			1:500	Thermo	A11008
Hoechst33342			1:1000 (IF)	Thermo	H1399

Supplementary table 4: List of qPCR primers used in this study. Given is the primer name, company name (if purchased, own refers to self designed primers) and the primer sequence or order number if purchased.

**RT-PCR primers**	**Company**	**Sequence or order#**
HsMYH6-FW-1	own	GTCAAAGCCAAGATTTTGTCCC
HsMYH6-RV-1	own	TCCTCAATCTTGTCGAACTTGG
HsMYH7-FW-2	own	TCTGTCCTGCTCTGTGTCTTT
HsMYH7-RV-2	own	CAAAGACTGCCATCTCCGAAT
HsNPPA-FW-13	own	GGGCAGGATGGACAGGATT
HsNPPA-RV-13	own	GGAGCCTCTTGCAGTCTGT
HsNPPB-FW-19	own	CTCCTGCTCTTCTTGCATCTG
HsNPPB-RV-19	own	GTTTGCCCTGCAAATGGTTG
HsMEF2A-FW-10	own	GCCCTTCAAGGCTTCAACTC
HsMEF2A-RV-10	own	CCTGAGATAACTGCCCTCC
HsATP2A2-FW-5	own	CATCTTCCAGATCACACCGC
HsATP2A2-RV-6	own	CTCCAGTATTGCAGGTTCCAG
HsCRISPLD1_Exon2+3_2	own	AGGTGTATCCAACAGCCTCTA
CRISPLD1_Ex2-3_Isoform1	own	CCCAGTGTGCTCCCAAATTC
HsCRISPLD1FW	own	TGC CCA AGA GTA TAC TGT CCT
HsCRISPLD1RV	own	GAT TTC GAA CCA CTC CAG CA
HsCRISPLD1_F2	own	GTC ACA AGT CCA TGA CAC CCA
HsCRISPLD1B9_KO_R	own	CTA TCC AAA CAG CCA GCA GGA
Hs18SFW	own	GAC ACG GAC AGG ATT GAC AG
Hs18SRV	own	CTA GTT AGC ATG CCA GAG TCT C
HsGAPDHFW	own	GTT CGT CAT GGG TGT GAA CC
HsGAPDHRV	own	GGT CAT GAG TCC TTC CAC GA
CRISPLD1	Qiagen	PPH20603A
RT² qPCR Primer Assay for Human SCN5A	Qiagen	PPH01671F
RT² qPCR Primer Assay for Human ADCY3	Qiagen	PPH02837A
RT² qPCR Primer Assay for Human ADRB1	Qiagen	PPH02091B
RT² qPCR Primer Assay for Human AGTR1	Qiagen	PPH02362F
RT² qPCR Primer Assay for Human ATP1A2	Qiagen	PPH09011A
RT² qPCR Primer Assay for Human CACNA2D3	Qiagen	PPH20424B
RT² qPCR Primer Assay for Human CACNG4	Qiagen	PPH17057A
RT² qPCR Primer Assay for Human RYR2	Qiagen	PPH10458A

Supplementary Literature:

1. Akhavan A (2010) Motorized traffic of a cardiac ion channel: implication of conventional kinesin in transport of Kv1.5 channels to the plasma membrane. J Physiol 588:903–904. doi: 10.1113/jphysiol.2009.186692
2. Andenæs K, Lunde IG, Mohammadzadeh N, Dahl CP, Aronsen JM, Strand ME, Palmero S, Sjaastad I, Christensen G, Engebretsen KVT, Tønnessen T (2018) The extracellular matrix proteoglycan fibromodulin is upregulated in clinical and experimental heart failure and affects cardiac remodeling. PLoS ONE 13. doi: 10.1371/journal.pone.0201422
3. Arnett DK, Devereux RB, Rao DC, Li N, Tang W, Kraemer R, Claas SA, Leon JM, Broeckel U (2009) Novel genetic variants contributing to left ventricular hypertrophy: the HyperGEN study. J Hypertens 27:1585–1593. doi: 10.1097/HJH.0b013e32832be612
4. Aubert G, Martin OJ, Horton JL, Lai L, Vega RB, Leone TC, Koves T, Gardell SJ, Krüger M, Hoppel CL, Lewandowski ED, Crawford PA, Muoio DM, Kelly DP (2016) The Failing Heart Relies on Ketone Bodies as a Fuel. Circulation 133:698–705. doi: 10.1161/CIRCULATIONAHA.115.017355
5. Bie Z-D, Sun L-Y, Geng C-L, Meng Q-G, Lin X-J, Wang Y-F, Wang X-B, Yang J (2016) MiR-125b regulates SFRP5 expression to promote growth and activation of cardiac fibroblasts. Cell Biol Int 40:1224–1234. doi: 10.1002/cbin.10677
6. Bullard TA, Protack TL, Aguilar F, Bagwe S, Massey HT, Blaxall BC (2008) Identification of Nogo as a novel indicator of heart failure. Physiol Genomics 32:182–189. doi: 10.1152/physiolgenomics.00200.2007
7. Cheng Y-S, Tang Y-Q, Dai D-Z, Dai Y (2012) AQP4 knockout mice manifest abnormal expressions of calcium handling proteins possibly due to exacerbating pro-inflammatory factors in the heart. Biochem Pharmacol 83:97–105. doi: 10.1016/j.bcp.2011.10.006
8. Chiamvimonvat N, Song L (2018) LRRC10 (Leucine-Rich Repeat Containing Protein 10) and REEP5 (Receptor Accessory Protein 5) as Novel Regulators of Cardiac Excitation-Contraction Coupling Structure and Function. J Am Heart Assoc Cardiovasc Cerebrovasc Dis 7. doi: 10.1161/JAHA.117.008260
9. Del Greco M F, Pattaro C, Luchner A, Pichler I, Winkler T, Hicks AA, Fuchsberger C, Franke A, Melville SA, Peters A, Wichmann HE, Schreiber S, Heid IM, Krawczak M, Minelli C, Wiedermann CJ, Pramstaller PP (2011) Genome-wide association analysis and fine mapping of NT-proBNP level provide novel insight into the role of the MTHFR-CLCN6-NPPA-NPPB gene cluster. Hum Mol Genet 20:1660–1671. doi: 10.1093/hmg/ddr035
10. Fan J, Qiu L, Shu H, Ma B, Hagenmueller M, Riffel JH, Meryer S, Zhang M, Hardt SE, Wang L, Wang DW, Qiu H, Zhou N (2018) Recombinant frizzled1 protein attenuated cardiac hypertrophy after myocardial infarction via the canonical Wnt signaling pathway. Oncotarget 9:3069–3080. doi: 10.18632/oncotarget.23149
11. Garg V, Muth AN, Ransom JF, Schluterman MK, Barnes R, King IN, Grossfeld PD, Srivastava D (2005) Mutations in NOTCH1 cause aortic valve disease. Nature 437:270–274. doi: 10.1038/nature03940
12. Jin X, Guo B, Yan J, Yang R, Chang L, Wang Y, Miao C, Liu S, Zhang H, Li Y (2015) Angiotensin II increases secreted frizzled-related protein 5 (sFRP5) expression through AT1 receptor/Rho/ROCK1/JNK signaling in cardiomyocytes. Mol Cell Biochem 408:215–222. doi: 10.1007/s11010-015-2497-9
13. Kim K-H, Antkiewicz DS, Yan L, Eliceiri KW, Heideman W, Peterson RE, Lee Y (2007) Lrrc10 is required for early heart development and function in zebrafish. Dev Biol 308:494–506. doi: 10.1016/j.ydbio.2007.06.005

14. Laeremans H, Hackeng TM, van Zandvoort MAMJ, Thijssen VLJL, Janssen BJA, Ottenheijm HCJ, Smits JFM, Blankesteijn WM (2011) Blocking of frizzled signaling with a homologous peptide fragment of wnt3a/wnt5a reduces infarct expansion and prevents the development of heart failure after myocardial infarction. Circulation 124:1626–1635. doi: 10.1161/CIRCULATIONAHA.110.976969
15. Lambert J-P, Ivosev G, Couzens AL, Larsen B, Taipale M, Lin Z-Y, Zhong Q, Lindquist S, Vidal M, Aebersold R, Pawson T, Bonner R, Tate S, Gingras A-C (2013) Mapping differential interactomes by affinity purification coupled with data-independent mass spectrometry acquisition. Nat Methods 10:1239–1245. doi: 10.1038/nmeth.2702
16. Lu B, Yu H, Zwartbol M, Ruifrok WPT, Gilst WH van, Boer RA de, Silljé HWW (2012) Identification of hypertrophy- and heart failure-associated genes by combining in vitro and in vivo models. Physiol Genomics 44:443–454. doi: 10.1152/physiolgenomics.00148.2011
17. Man J, Barnett P, Christoffels VM (2018) Structure and function of the Nppa–Nppb cluster locus during heart development and disease. Cell Mol Life Sci 75:1435–1444. doi: 10.1007/s00018-017-2737-0
18. Montpetit ML, Stocker PJ, Schwetz TA, Harper JM, Norring SA, Schaffer L, North SJ, Jang-Lee J, Gilmartin T, Head SR, Haslam SM, Dell A, Marth JD, Bennett ES (2009) Regulated and aberrant glycosylation modulate cardiac electrical signaling. Proc Natl Acad Sci U S A 106:16517–16522. doi: 10.1073/pnas.0905414106
19. Nishihama N, Nagayama T, Makino S, Koishi R (2019) Mice lacking fat storage-inducing transmembrane protein 2 show improved profiles upon pressure overload-induced heart failure. Heliyon 5:e01292. doi: 10.1016/j.heliyon.2019.e01292
20. Sato M, Tsai H-J, Yost HJ (2006) Semaphorin3D regulates invasion of cardiac neural crest cells into the primary heart field. Dev Biol 298:12–21. doi: 10.1016/j.ydbio.2006.05.033
21. Takefuji Mikito, Wirth Angela, Lukasova Martina, Takefuji Seiko, Boettger Thomas, Braun Thomas, Althoff Till, Offermanns Stefan, Wettschureck Nina (2012) G13-Mediated Signaling Pathway Is Required for Pressure Overload–Induced Cardiac Remodeling and Heart Failure. Circulation 126:1972–1982. doi: 10.1161/CIRCULATIONAHA.112.109256
22. Tarazón E, Roselló-Lletí E, Rivera M, Ortega A, Molina-Navarro MM, Triviño JC, Lago F, González-Juanatey JR, Orosa P, Montero JA, Salvador A, Portolés M (2014) RNA Sequencing Analysis and Atrial Natriuretic Peptide Production in Patients with Dilated and Ischemic Cardiomyopathy. PLoS ONE 9. doi: 10.1371/journal.pone.0090157
23. Wang F-F, Ha L, Yu H-Y, Mi L, Han J-L, Gao W (2017) Altered serum level of cartilage oligomeric matrix protein and its association with coronary calcification in patients with coronary heart disease. J Geriatr Cardiol JGC 14:87–92. doi: 10.11909/j.issn.1671-5411.2017.02.002
24. Wang JJ-C, Rau C, Avetisyan R, Ren S, Romay MC, Stolin G, Gong KW, Wang Y, Lusis AJ (2016) Genetic Dissection of Cardiac Remodeling in an Isoproterenol-Induced Heart Failure Mouse Model. PLoS Genet 12. doi: 10.1371/journal.pgen.1006038
25. van Weeghel M, te Brinke H, van Lenthe H, Kulik W, Minkler PE, Stoll MSK, Sass JO, Janssen U, Stoffel W, Schwab KO, Wanders RJA, Hoppel CL, Houten SM (2012) Functional redundancy of mitochondrial enoyl-CoA isomerases in the oxidation of unsaturated fatty acids. FASEB J Off Publ Fed Am Soc Exp Biol 26:4316–4326. doi: 10.1096/fj.12-206326
26. Yang G, Chen S, Ma A, Lu J, Wang T (2017) Identification of the difference in the pathogenesis in heart failure arising from different etiologies using a microarray dataset.

Clinics 72:600–608. doi: 10.6061/clinics/2017(10)03
27. Yu B, Zheng Y, Alexander D, Manolio TA, Alonso A, Nettleton JA, Boerwinkle E (2013) Genome-Wide Association Study of a Heart Failure-Related Metabolomic Profile among African Americans in the Atherosclerosis Risk in Communities (ARIC) Study. Genet Epidemiol 37:840–845. doi: 10.1002/gepi.21752
28. Zhang Y, Bilbao A, Bruderer T, Luban J, Strambio-De-Castillia C, Lisacek F, Hopfgartner G, Varesio E (2015) The Use of Variable Q1 Isolation Windows Improves Selectivity in LC-SWATH-MS Acquisition. J Proteome Res 14:4359–4371. doi: 10.1021/acs.jproteome.5b00543
29. Zhu X, Wang H, Liu F, Chen L, Luo W, Su P, Li W, Yu L, Yang X, Cai J (2013) Identification of micro-RNA networks in end-stage heart failure because of dilated cardiomyopathy. J Cell Mol Med 17:1173–1187. doi: 10.1111/jcmm.12096
